# Feedback inhibition of cAMP effector signaling by a chaperone-assisted ubiquitin system

**DOI:** 10.1038/s41467-019-10037-y

**Published:** 2019-06-12

**Authors:** Laura Rinaldi, Rossella Delle Donne, Bruno Catalanotti, Omar Torres-Quesada, Florian Enzler, Federica Moraca, Robert Nisticò, Francesco Chiuso, Sonia Piccinin, Verena Bachmann, Herbert H Lindner, Corrado Garbi, Antonella Scorziello, Nicola Antonino Russo, Matthis Synofzik, Ulrich Stelzl, Lucio Annunziato, Eduard Stefan, Antonio Feliciello

**Affiliations:** 1 Department of Molecular Medicine and Medical Biotechnologies, University Federico II, 80131 Naples, Italy; 2 Department of Pharmacy, University Federico II, 80131 Naples, Italy; 3 Institute of Biochemistry and Center for Molecular Biosciences, University of Innsbruck, A-6020 Innsbruck, Austria; 4 Department of Chemical Sciences, University Federico II, 80131 Naples, Italy; 5 European Brain Research Institute, Rita Levi-Montalcini Foundation and Department of Biology, University Tor Vergata, 00143 Rome, Italy; 6 Division of Clinical Biochemistry, Biocenter Medical University of Innsbruck, Innrain 80-82, A-6020 Innsbruck, Austria; 7 Department of Neuroscience, Reproductive and Odontostomatological Sciences, University Federico II, 80131 Naples, Italy; 8 I.R.C.S., BIOGEM, Ariano Irpino, 83031 Avellino, Italy; 9 Department of Neurodegeneration, Hertie Institute for Clinical Brain Research (HIH), University of Tübingen and German Center for Neurodegenerative Diseases (DZNE), 72076 Tübingen, Germany; 10 Institute of Pharmaceutical Sciences, University of Graz and BioTechMed-Graz, 8010 Graz, Austria; 11 IRCCS SDN, 80143 Naples, Italy

**Keywords:** Cell signalling, Ubiquitin ligases, Ubiquitylation

## Abstract

Activation of G-protein coupled receptors elevates cAMP levels promoting dissociation of protein kinase A (PKA) holoenzymes and release of catalytic subunits (PKAc). This results in PKAc-mediated phosphorylation of compartmentalized substrates that control central aspects of cell physiology. The mechanism of PKAc activation and signaling have been largely characterized. However, the modes of PKAc inactivation by regulated proteolysis were unknown. Here, we identify a regulatory mechanism that precisely tunes PKAc stability and downstream signaling. Following agonist stimulation, the recruitment of the chaperone-bound E3 ligase CHIP promotes ubiquitylation and proteolysis of PKAc, thus attenuating cAMP signaling. Genetic inactivation of CHIP or pharmacological inhibition of HSP70 enhances PKAc signaling and sustains hippocampal long-term potentiation. Interestingly, primary fibroblasts from autosomal recessive spinocerebellar ataxia 16 (SCAR16) patients carrying germline inactivating mutations of CHIP show a dramatic dysregulation of PKA signaling. This suggests the existence of a negative feedback mechanism for restricting hormonally controlled PKA activities.

## Abstract (web summary)

How intracellular cAMP activate PKA is well-characterized, but PKA inactivation remains poorly understood. Here, Rinaldi et al. show that CHIP/HSP70 ubiquitinates the catalytic subunit of PKA, with implications for the human disease spinocerebellar ataxia 16, as patients often have CHIP mutations.

## Introduction

Many hormones and neurotransmitters operate through cell membrane localized G-protein coupled receptors (GPCR) to transmit messages through compartmentalized signaling pathways inside of the target cells^1–3^. Activation of adenylate cyclase by a given GPCR ligand increases intracellular levels of the second messenger cAMP, which in turn activates protein kinase A (PKA). The inactive PKA holoenzyme is composed of two regulatory (R) and two catalytic (PKAc, C) subunits. The binding of cAMP to R dissociates the tetrameric holoenzyme, releasing free active PKAc subunits which phosphorylate a wide array of cellular substrates, regulating fundamental aspects of cell physiology^4,5^. In mammalian cells, PKA is concentrated in membranes and subcellular compartments through binary interactions with a collection of A-kinase anchoring proteins (AKAPs)^6–9^. As consequence of PKA targeting by AKAPs, cAMP signals generated at cell membranes propagate from sites of signal generation to distal compartments. This modular signaling system efficiently couples ligand-mediated activation of membrane receptors to the spatio-temporal mobilization of cAMP fluxes which are sensed by PKA and relayed to downstream effectors^10–15^. Propagation of PKAc signaling throughout the cell is followed by a precisely controlled refractory phase at different stages of the pathway to uncouple the hormonal input signal from continuous mobilization of cAMP^16^. Among others, this period is characterized by a distinct reduction of PKAc levels attributed to reduced ribosome translation or stability of the kinase subunit^17,18^. Activation of phosphodiesterases (PDE) counteracts cAMP levels and control the duration and amplitude of downstream signaling^19,20^. The binding of the endogenous protein kinase inhibitor (PKI) to PKAc also contributes to the attenuation of cAMP signaling in hormone-stimulated cells^21,22^.

The ubiquitin-proteasome system (UPS) plays a major role in many aspects of cell growth, survival and metabolism. Post-translational modification by covalently attaching multiple ubiquitin molecules target the cargo protein for the UPS-mediated proteasomal degradation^23^.By modulating the levels of target proteins, the UPS controls essential aspects of cell biology. Proteolysis of components of the cAMP cascade at different stages emerged as a relevant mechanism to modulate the extent and duration of the cAMP-activated PKA response^24,25^. An integrated network between the UPS and components of the cAMP effector pathway finely controls the extent and duration of GPCR-generated signaling, placing the ubiquitin system at a center stage of cellular hormone action. PKA is regulated by the UPS. An example is the E3 ubiquitin-ligase praja2 which targets PKA R subunits for degradation and acts as scaffold to anchor the PKA holoenzyme to specific subcellular compartments. Co-localization of praja2•PKA complexes with PKA substrates contributes to efficient integration and propagation of cAMP signals to specific target sites. Following GPCR activation, praja2 couples ubiquitylation to proteolysis of the R subunits of PKA, thereby sustaining PKAc phosphotransferase activities and downstream signaling^26^.

Recent evidence indicates that PKAc undergoes proteolysis through a lysosomal pathway. Thus, the binding to the Rho GTPase-activating protein (RhoGAP) family member ARHGAP36 promotes ubiquitylation of PKAc at K286 and its subsequent targeting to degradation by lysosomal cargos. The peculiar localization of ARHGAP36 at endosomal membranes and the finding that phosphorylation of the cAMP-responsive element binding protein (CREB) was unaffected in cells depleted of ARHGAP36 suggesting that ARHGAP36 is involved in the degradation of a restricted pool of PKAc, mainly contributing to PKA signaling within the endosomal compartment^27^. In addition, the tissue-restricted expression of ARHGAP36 compared to broad expression profiles of PKAc in practical almost every mammalian cell and tissue suggests that other mechanisms exist that contribute to control PKAc stability upon generation of cAMP fluxes. So far, the regulation of PKAc by the UPS and its involvement in the feedback control mechanisms underlying the hormone action were largely unknown.

Here, we identify a chaperone-mediated mechanism that finely couples cAMP oscillations to ubiquitylation and proteolysis of PKAc, controlling the extent and duration of downstream signaling. Deregulation of this control system in SCAR16 patients highlights a critical function of the cAMP-UPS interlinkage in a pathological setting.

## Results

### cAMP promotes ubiquitylation and proteolysis of PKAc

**Fig. 1:**
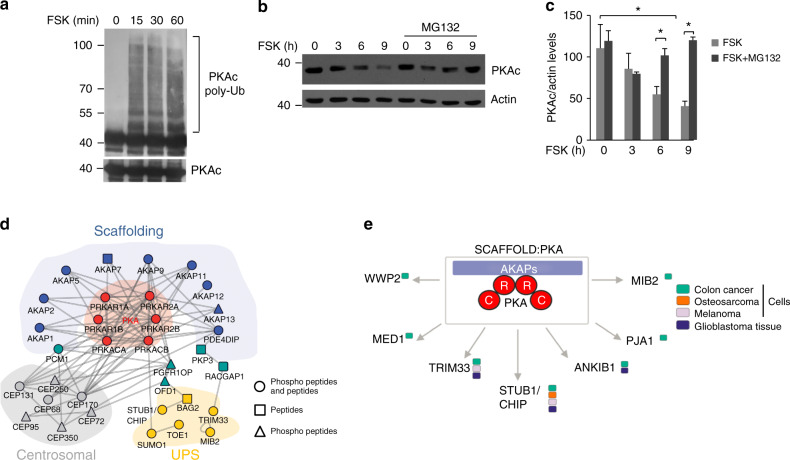
cAMP stimulation induces ubiquitylation and proteolysis of PKAc. **a** Cells were transiently transfected with HA-ubiquitin. Twenty-four hour after transfection, cells were treated with MG132 (20 µM) for 1 h and then stimulated with FSK (40 μM) for the indicated time points. Lysates were subjected to immunoprecipitation with anti-PKAc. The precipitates were immunoblotted with anti-PKAc and anti-HA antibodies. **b** HEK293 cells were serum deprived overnight and then stimulated with forskolin (FSK 40 μM). Where indicated, cells were pretreated with the proteasome inhibitor MG132. Cells were, then, harvested at the indicated time points and lysed. The lysates were immunoblotted with the indicated antibodies. **c** Quantitative analysis of the experiments shown in **b**. A mean value ± S.E.M. of four independent experiments is reported; Student’s *t* test, *p < 0.05. **d** Illustrated are selected protein interactions of macromolecular PKA complexes following affinity purifications from a collection of cancer cells and glioblastoma tissue. Following gene ontology (GO) annotations, the whole dataset was used to extract functionally linked complexes (color coded). Selected subnetworks linked to PKA complex formation, kinase scaffolding, centrosome function, and the ubiquitin-proteasome system are depicted. The shape of listed icons indicates mode of identification in the MS/MS analyses. **e** Shown are proteins from the UPS identified upon affinity isolations of endogenous PKA complexes from different cell/tissue sources

To address the role of UPS in PKA signaling, we monitored ubiquitylation and stability of PKAc in cells stimulated with forskolin (FSK), a diterpene that activates adenylate cyclase. The rise in cAMP levels rapidly and markedly induced ubiquitylation of endogenous PKAc (Fig. 1a). Importantly, ubiquitylation of PKAc induced by cAMP initiated a time-dependent decline of PKAc levels (Fig. 1b, c). Inhibiting proteasome activity with MG132 prevented PKAc degradation, indicating that the proteasome mediates the decline of PKAc levels in FSK-stimulated cells. These data suggested the presence of an E3 ub-ligase responsible for PKAc ubiquitylation upon cAMP mobilization.

To identify the E3 ub-ligase involved in the cAMP-triggered desensitization of PKAc activity, we performed proteomic based analyses of affinity-isolated PKA holoenzyme complexes from several cells lines and human glioblastoma tissue. We applied PKA-selective Rp-8-AHA-cAMP-agarose as affinity matrix. PKA R subunits bind immobilized Rp-8-cAMP-analogs largely without immediate dissociation of the R:PKAc complexes. As a negative-control experiment, we added excess of cAMP to the lysates to mask the cAMP-binding sites in the PKA R subunits for binding to the cAMP agarose^8,28^. We enriched PKA subunits and significant amounts of known interacting proteins such as AKAPs (e.g., AKAPs 1, 2, 5, 7, 9, 11–13). Shown in an excerpt of the whole PKA network focusing on existing links to the UPS and interlinked centrosomal complexes (Fig. 1d). Our data identified the E3 ubiquitin-ligase CHIP (C-terminus of HSP70 interacting protein; gene name Stub1) as a central component of macromolecular PKA complexes in the tested cell settings. We determined the proteomic composition of affinity-isolated PKA complexes from different sources and identified association of CHIP in osteosarcoma cells, melanoma cells, different colon cancer cell types and glioblastoma tissue. The summary of identified UPS connections to PKA complexes in the different cell types are depicted in Fig. 1e. More precisely, we identified interaction links of PKA to STUB1 in A375 melanoma cells, in KM12 and SW480 colon cancer cells, in osteosarcoma cells (U2OS) and in the only tested patient tissue (glioblastoma).

CHIP is an E3 ubiquitin ligase widely expressed in mammalian cells and tissues^29,30^. CHIP acts as an HSP90/70-assisted E3 ligase primarily involved in protein quality control. CHIP pathway dysregulation has been observed in different types of cancer, aging pathways, and neurodegenerative disorders^31–34^.

**Fig. 2:**
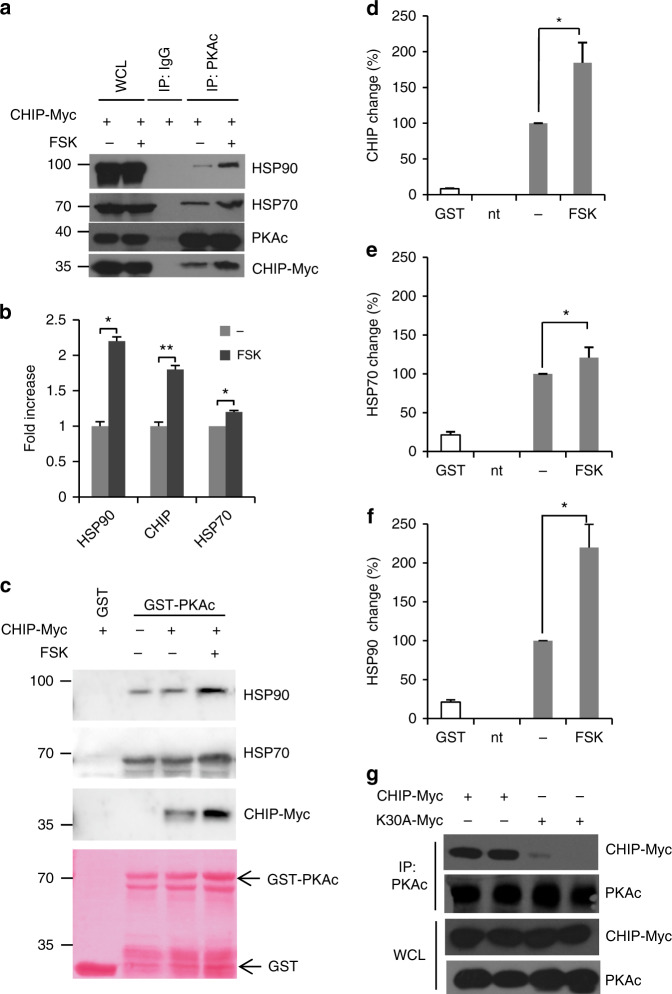
Recruitment of CHIP/HSPs complex to PKAc. **a** HEK293 cells were transiently transfected with CHIP-Myc vector, treated for 30 min with FSK and lysed. Lysates were immunoprecipitated with anti-PKAc antibody. The precipitates and an aliquot of lysates were immunoblotted with anti-HSP70/90, anti-PKAc and anti-myc antibodies. **b** Quantitative analysis of the experiments shown in **a**. A mean value ± S.E.M. of three independent experiments is reported. Student’s *t* test, *p < 0.05. **c** GST-PKAc pull-down experiments from HEK293 lysates expressing CHIP-myc and HSP70-V5. Ponceau staining (lower panel) and immunoblot analysis of HSP90, CHIP and HSP70 are shown. **d**–**f** Quantification levels were normalized against the basal level of the nontreated GST-PKAc samples (nt nontransfected cells). Representative results of five independent experiments (mean ± SEM; statistical test: ANOVA, *p < 0.05). **g** Lysates from cells transiently transfected with CHIP-myc vector (wild type or K30A mutant) were subjected to immunoprecipitation using Rluc specific antibodies. Precipitates and an aliquot of lysates were immunoblotted with the indicated antibodies

We now asked if CHIP interacts with PKAc. As shown in Fig. 2a, a complex composed of PKAc and CHIP can be isolated from whole cell lysates. CHIP-associated chaperones (HSP70/90) were also present in the PKAc immunoprecipitates. The association between CHIP, HSP90/70, and PKAc is inducible by FSK treatment (Fig. 2a, b). Similar findings were replicated with exogenous, overexpressed CHIP and HSP70 (Supplementary Fig. 1). We also evaluated the interaction between PKAc and CHIP in vitro by GST pull-down experiments. These results, shown in Fig. 2c, d, demonstrated that recombinant PKAc binds CHIP, and that the interaction is induced upon cAMP elevation. Similarly, HSP90, and to a lesser extent HSP70, could be recruited into the GST-PKAc/CHIP complex by cAMP stimulation with FSK (Fig. 2c, e, f). CHIP contains a tetra-tricopeptide (TPR) motif tandem repeats at its N-terminus that mediates interaction with HSP70, whereas the C-terminal segment includes a U-box domain separated by a charged coiled-coil region^35^. A CHIP mutant (CHIP-K30A) lacking the HSP70 binding activity failed to interact with PKAc, supporting the role of HSP70 in mediating the interaction of PKAc to CHIP (Fig. 2g).

**Fig. 3:**
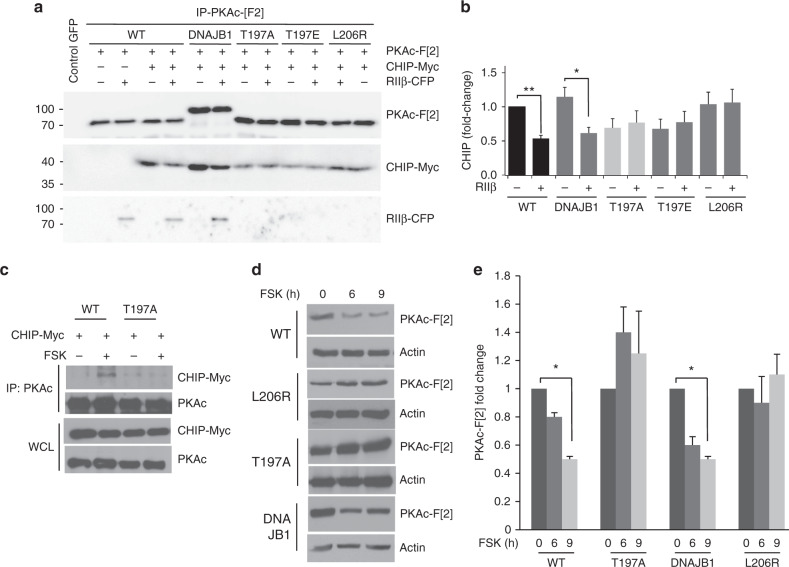
cAMP regulation of PKAc:CHIP complex. **a** Impact of the PKAc mutation on CHIP binding. Cells co-transfected with RIIβ-CFP, CHIP-myc and several mutants of PKAc fused to the *R*luc PCA Fragment 2 (PKAc-[F2]) were lysed and subjected to immunoprecipitation using Rluc 4400 antibodies. Immunocomplexes were immunoblotted with the indicated antibodies. **b** Densitometric evaluation of the Fig. 3a signals. Statistical analysis of five independent experiments is shown. Mean ± SEM. Student’s *t* test,, *p < 0.05, **p ≤ 0.01). **c** Cells transiently transfected with the indicated PKAc vectors were left untreated or stimulated with FSK. Lysates were immunoprecipitated with Rluc 4410 specific antibody and immunoblotted with anti-PKAc and anti-Myc antibodies. **d** Cells transiently transfected with the indicated PKAc vectors were left untreated or stimulated with FSK. Lysates were immunoblotted with anti-PKAc and anti Actin antibodies. **e** Quantitative analysis of the experiments shown in **d**. A mean value ± S.E.M of three independent experiments is shown. Student’s *t* test, *p < 0.05

Recently, mutations in PKAc linked to different disease states have been identified. In Cushing’s syndrome, a disease characterized by excess glucocorticoid production from tumors and hyperplasia of the adrenal cortex, is associated with activating PKAc mutations, such as L206R, and gene amplifications^36–39^. Further, PKAc fusions have been described in fibrolamellar hepatocellular carcinoma. Fusion of the molecular chaperone DnaJ with the PKAc gene of the Cα subunit (DNAJB1) induces carcinogenesis^40,41^. We asked if these PKAc variants interact with CHIP. We show that all tested PKAc variants formed complexes with CHIP (Fig. 3a). However, interaction experiments with CHIP in the presence of coexpressed RIIβ showed differences in complex formation. RIIβ interacted, as predicted, with wild type PKAc and with the DNAJB1–PKAc fusion. Formation of the holoenzyme complexes significantly reduced interaction with CHIP (Fig. 3a, b). In contrast, two PKAc mutants (L206R and T197A/E), did not form complexes with overexpressed RIIβ (Fig. 3a, b) and their binding to CHIP could not be competed by coexpressed RIIβ. In studies with a *Renilla* luciferase (*R*luc) based protein-fragment complementation assay (PCA), we previously showed that L206R does not form PKA holoenzyme^42^. In Supplementary Fig. 2, we validated these findings by the *R*luc PCA experiments and also showed that a mutation in the kinase activation loop (T197A), which is located in the R:PKAc binding interface, abrogates PKA complex formation. We then tested the role of T197 in binding to CHIP. Figure 3c shows that T197A also blocked cAMP-induced PKAc binding to CHIP. Next, we focused on the FSK induced turnover of PKAc. This data supports the idea that PKAc released from holoenzyme following cAMP stimulation interacts with and is removed by the CHIP–UPS pathway. We next assessed the stability of PKAc variants in cells stimulated with cAMP. Both, T197A and L206R PKAc variants were resistant to degradation induced by cAMP stimulation (Fig. 3d, e). In contrast, the DNAJB1 fusion was sensitive to proteolysis induced by FSK stimulation (Fig. 3d, e).

**Fig. 4:**
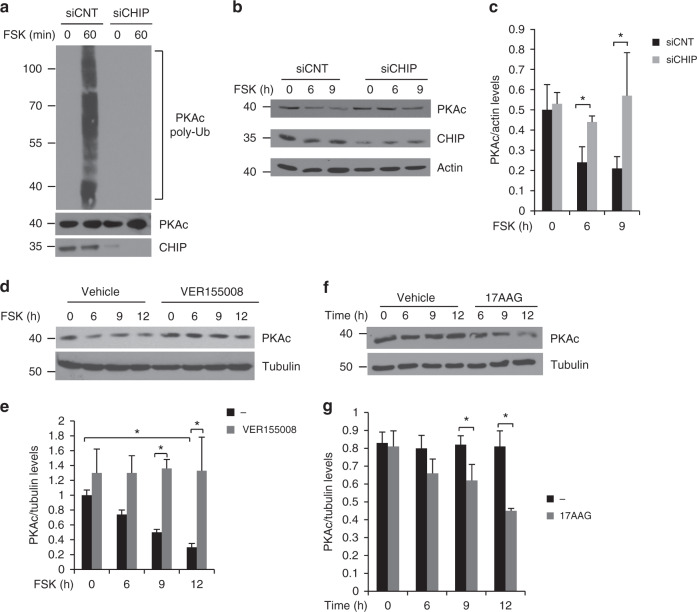
CHIP ubiquitylates PKAc. **a** Lysates from cells transiently transfected with HA-ubiquitin and siRNAs (control or siRNA targeting CHIP), left untreated or stimulated with FSK, were immunoprecipitated with anti-PKAc. The precipitates were immunoblotted with anti-PKAc and anti-HA antibodies. **b** Cells transfected with siRNAs (control or siRNA targeting CHIP) were serum deprived overnight and treated with FSK. Lysates were immunoblotted with the indicated antibodies. **c** Quantitative analysis of the experiments shown in **b**. A mean value ± S.E.M. of three independent experiments is reported. Student’s *t* test, *p < 0.05. **d** Cells were stimulated with FSK, either in absence or presence of the HSP70 inhibitor (VER155008). Lysates were immunoblotted for PKAc and tubulin. **e** Quantitative analysis is shown. A mean value ± S.E.M. of three independent experiments is reported. Student’s *t* test, *p < 0.05. **f** Cells were untreated (vehicle) or treated with HSP90 inhibitor (17AAG) and harvested at the indicated times. Lysates were immunoblotted for PKAc and tubulin. **g** Quantitative analysis is shown. A mean value ± S.E.M. of three independent experiments is reported. Student’s *t* test, *p < 0.05

Next, we analyzed the role of CHIP/HSPs complexes in the control of PKAc ubiquitylation and stability. Silencing of CHIP (Fig. 4a) or expression of CHIP-K30A mutant (Supplementary Fig. 3) abrogated PKAc ubiquitylation after FSK stimulation. We then asked if CHIP controls PKAc levels. As shown in Fig. 4b, c, CHIP knockdown prevented PKAc decline following holoenzyme dissociation. CHIP ubiquitinylates HSP70-bound substrates.As expected, pretreatment of cells with the specific HSP70 inhibitor (VER155008) also stabilized free PKAc (Fig. 4d, e). In contrast, HSP90 inhibition with 17AAG decreased PKAc levels as a function of time after addition of the inhibitor, even in the absence of FSK. These results suggest that HSP70 is required for CHIP degradation of PKAc, where HSP90 protects the PKAc subunit from proteolysis (Fig. 4f, g). A similar mechanism has been proposed for other members of the AGC kinase family; HSP90 binding protects the newly translated kinases from ubiquitin-independent proteolysis^43,44^.

**Fig. 5:**
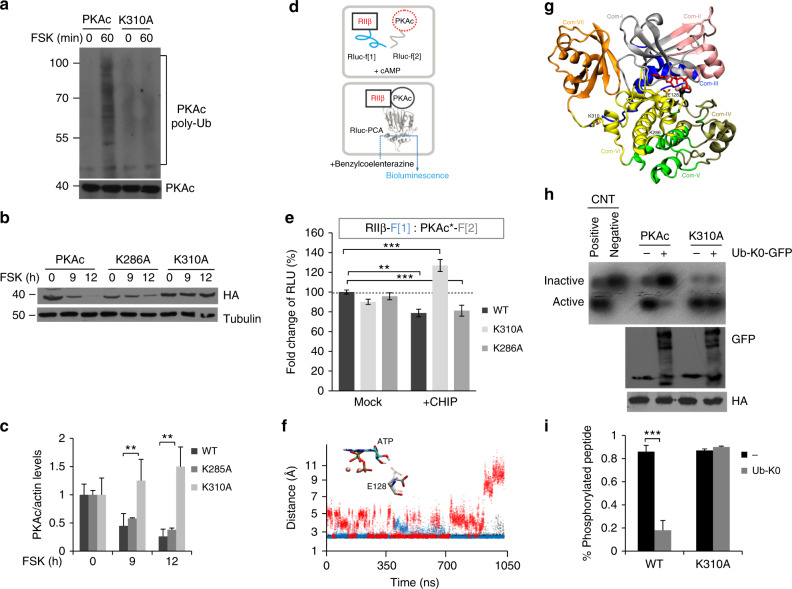
Ubiquitylation of K310 affects PKAc dynamics and activity. **a** Cells were transiently co-transfected with myc-tagged PKAc construct (either wild type or K310A mutant) and HA-ubiquitin. Twenty-four hour after transfection, cells were treated with MG132 (20 µM) for 1 h and then stimulated with FSK (40 μM/60 min). Lysates were subjected to immunoprecipitation with anti-PKAc, and immunoblotted with anti-PKAc and anti-HA antibodies. **b** Cells transfected with HA-tagged PKAc vectors (wild type, K286A or K310A) were serum deprived overnight and stimulated with FSK (40 μM). **c** Quantitative analysis of the experiments shown in **b**. A mean value ± S.E.M. of three independent experiments is reported (statistical test: ANOVA, **p < 0.05). **d, e** PPI reporter analyses of transiently coexpressed *R*luc PCA reporter constructs in the presence or absence of myc-tagged CHIP (ratio of transfection 1:1:2). Quantification of five independent experiments (mean ± SEM) are shown. Student’s two-tailed *t* test was used to evaluate statistical significance. Confidence level: ^**^P < 0.01, ^***^P < 0.001. **f** Time evolution of distances between the carboxyl group of E128 and the 2′ hydroxyl of ATP ribose in free (black), K286-Ub (blue) and K310-Ub (red) PKAc and licorice representation of the conformational switch observed for E128. **g**. Community maps retrieved by the dynamical network analysis for K310-Ub. Communities color code is detailed in Supplementary Tables 2 and 3. ATP is depicted in red stick, and Mg^++^ ions are depicted in pink spheres. The Girvan–Newman algorithm split the dynamic network of K310-Ub PKAc into seven communities (from Com-I to Com-VII). **h** Cells were transfected with PKAc vectors (either wild type or the K310 mutant), in the presence or absence of Ub-K0-GFP mutant, stimulated with FSK (40 µM/30 min) to promote ubiquitylation of PKAc and lysed. Lysates were subjected to in vitro kinase assays. **i** PKAc activity is presented as the percentage of phosphorylated peptide calculated as the ratio between phosphorylated peptide signal and total signal. Data are expressed as mean value ± S.E.M. of four independent experiments. Student’s *t* test, ^***^P < 0.001. An aliquot of the lysates was subjected to immunoblot analysis with anti-HA (fo PKAc) and anti-GFP (Ub-K0)

We mapped the principal acceptor site for the ubiquitin moiety on PKAc by mass spectrometry (MS) analysis of affinity-isolated PKA isoforms (immunoprecipitation) or holoenzyme complexes (cAMP-precipitation). The MS-based analyses revealed besides ubiquitylated R subunits a single ubiquitylation site in PKAc, the lysine 310 (K310) respectively (Supplementary Fig. 4a). In Supplementary Fig. 4b, c, we indicate the theoretical masses of the fragment ions of the ubiquitinated peptide K(GG)VEAPFIPK of PKAc. The K310A mutation in PKAc significantly reduced FSK-induced ubiquitylation (Fig. 5a). Moreover, K310A was resistant to FSK-induced PKAc degradation (Fig. 5b, c). Previous studies demonstrated that ubiquitylation of K286 promotes PKAc proteolysis by a nonproteasomal pathway, involved in maintaining a basal turnover of PKAc by a cAMP-independent mechanisms and in absence of any stimuli^27^.

To determine the contribution of K286 ubiquitylation to PKAc stability during adenylate cyclase stimulation, we generated a PKAc mutant carrying a K286A mutation and tested its stability in cells stimulated with FSK. In contrast to K310A, cAMP-induced degradation of the K286A mutant to a similar extent as a wild-type construct (Fig. 5b, c). These findings indicate that K310 is the principal acceptor site of the ubiquitin moiety and constitutes the critical determinant of PKAc stability. To test the impact of both mutations on the formation of PKA type I and type II complexes, we subjected the Rluc PCA reporter system to quantify R:C complex formation. Upon CHIP coexpression, we observed that exclusively the K310A mutated PKA holoenzyme complexes are stabilized. It underlines the notion that both, PKAc subunits of type I and type II holoenzyme complexes are the target of the CHIP-centered UPS (Fig. 5d, e and Supplementary Fig. 5a). Using the *R*luc PCA reporter as readout for PPI, we could not detect any impact of CHIP coexpression on C:PKI complexes (Supplementary Fig. 5b).

### Ubiquitylation of K310 affects PKAc dynamics and activity

Recent computational studies on ZAP70 kinases revealed that ubiquitylation induced an allosteric modulation of their catalytic activity^45^. Accordingly, we asked whether ubiquitylation of PKAc affects kinase dynamics. We performed a molecular dynamics (MD) study of PKAc in complex with ATP and two Mg^2+^ ions with the single ubiquitin-bound at K310 (K310-Ub PKAc) and compared it with non ubiquitinated PKAc in complex with ATP and two Mg^2+^ ions (free-PKAc) or ubiquitinated at K286 (K286-Ub PKAc). Three independent simulations of 350 ns, for a total simulation time of 1.05 μs per system, were performed. The trajectory analysis of the K310-Ub PKAc system highlighted that, although the flexibility of the C-terminal tail allows a wide conformational range, Ub never impinged on the substrate-binding site (Supplementary Movies 1 and 2).

Analysis of K310-Ub trajectory revealed a weakening of ATP interactions in the C-lobe. E128 showed faster transitions between two rotamers, inducing a frequent breaking of the hydrogen bond between the carboxylic group of E128 and the 2’ hydroxyl group of the ATP ribose (Fig. 5f). This behavior, not observed in the free- or K286-Ub-MD simulations, was more pronounced in one MD run, where it led to the disruption of the ATP/2Mg^++^ binding mode. To confirm that the dynamics of ATP binding residues was influenced by K310 ubiquitylation, we performed a dynamical network and community map analysis using the Girvan–Newman algorithm^46^ on the MD simulations of K310-Ub-PKAc (Fig. 5g, Supplementary Table 1), free-PKAc (Supplementary Fig. 6a and Supplementary Table 2) and K286-Ub-PKAc (Supplementary Fig. 6b and Supplementary Table 3), and compared with those identified for the PKAc^ATP/2Mg++^ system^47^. We saw a decrease in the number of communities in ubiquitinated PKAc (K310-Ub and K286-Ub) with respect to the free PKAc, indicating a higher correlation of motions between residues in the C-lobe. In K310-Ub-PKAc the increased correlation also involved residues K310 and E128, which in the K310-Ub PKAc simulations were part of the same community (Com-VI). These residues in the free-PKAc, and in the K286-Ub systems split into two different communities (Com-6/Com-11 and Com-IV*/Com-VIII*, respectively) (Supplementary Fig. 6a, b). Similarly, the calculation of optimal and suboptimal paths performed with WISP^48^ showed a shift towards shorter lengths (i.e., more correlated motions) of paths connecting K310 and E128 in the K310-Ub PKAc MD simulations. In particular, the shortest path between K310 and E128 was K310:R309:E128 (Length: 1.66) (Supplementary Fig. 7**)**. The same path in the free-PKAc and in K286-Ub PKAc was ranked, respectively as the tenth and the seventh shortest suboptimal path (length: 2.06 and 2.02, respectively). In contrast, the optimal path in both the free and K286-Ub- PKAc consisted of K310:Y180:E128 (Length: 1.78 and 1.74, respectively) (Supplementary Fig. 7), which was the second shortest suboptimal path in the K310-Ub PKAc (length: 1.67). Taken together, the results of the analysis of correlated motions supported the hypothesis that K310 ubiquitylation affected the dynamic of E128 in K310-Ub, but not in the free or K286-Ub PKAc.

To prove that monoubiquitylation, indeed, affects PKAc activity, we took advantage of using an ubiquitin mutant protein (Ub-K0) which lacks the lysine residues at positions 6, 11, 27, 29, 33, 48, and 63 that are critical for ubiquitin chain elongation^49^. Cells were transiently co-transfected with Ub-K0 and PKAc vectors, either wild type or mutant K310A, stimulated with FSK for 1 h to induce ubiquitylation of PKAc, harvested and lysed. Cell lysates were subjected to in vitro PKA enzymatic assays. The results demonstrate that coexpression of Ub-K0 mutant dramatically inhibited the activity of wild-type PKAc (Fig. 5h, i). In contrast, Ub-K0 protein had no significant effects on the activity of the coexpressed K310A mutant (Fig. 5h, i). These results indicate that K310 monoubiquitylation acts as inhibitory mechanism of PKAc activity in course of cAMP stimulation.

### CHIP attenuates nuclear cAMP signaling

**Fig. 6:**
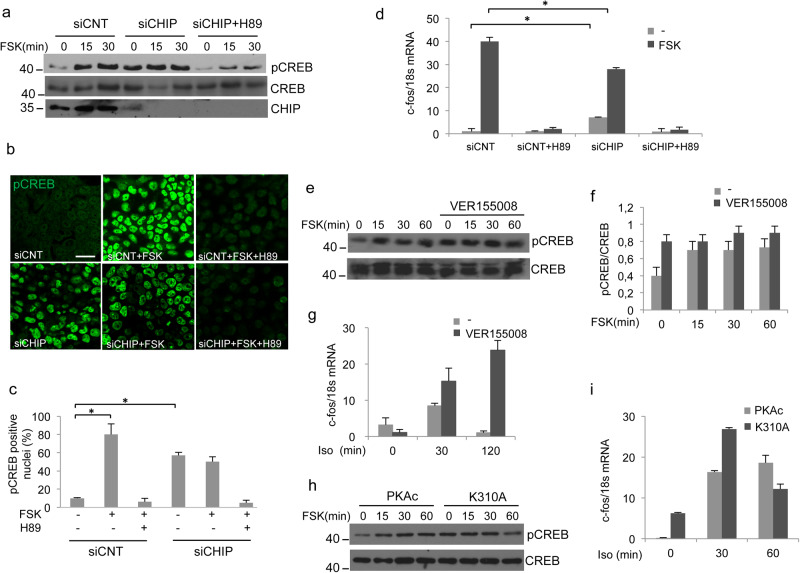
CHIP controls nuclear cAMP signaling. **a** Cells were transfected with siRNAs (control or siRNA targeting CHIP), serum deprived overnight and then stimulated with FSK. Where indicated, cells were pretreated for 1 h with the PKA inhibitor H89 (10 μm). Lysates were immunoblotted for phosphoSer133-CREB (pCREB), CREB and CHIP. **b** Same as in **a**, with the exception that cells were formalin-fixed and immunostained for pCREB antibodies. Representative confocal images are shown. Scale bar, 10 μm. **c** Quantitative analysis of the experiments shown in **b**. A mean value ± S.E.M. of three independent experiments is reported. A total of 100 cells were scored in each set of experiments. Student’s *t* test, *p < 0.05. **d** Quantitative PCR with reverse transcription showing *c*-fos accumulation in cells transfected with siRNAs (control or siRNA for CHIP). Before harvesting, cells were treated with FSK (40 μM/90 min). Where indicated, cells were pretreated with H89 (10 μM). The data represent a mean value ± S.E.M from three independent experiments made in duplicate. Student’s *t* test, *p < 0.05. **e**, **f** Cells were stimulated with FSK, either in absence or presence or VER155008. Lysates were immunoblotted with anti-pCREB and anti-CREB antibodies. A quantitative analysis of three independent experiments is reported as a mean value ± S.E.M. **g** *c*-fos accumulation in cells stimulated with isoproterenol (Iso) either in absence or presence or VER155008. The data represent a mean value ± S.E.M from three independent experiments made in duplicate. **h** Cells were transiently transfected with PKAc vectors (wild type and K310A), serum deprived overnight and then stimulated with FSK (40 μM). Lysates were immunoblotted for pCREB and CREB. **i** *c*-fos accumulation in cells transfected with PKAc vectors (wild type or K310A) and left untreated or stimulated with isoproterenol (Iso 10 μM). The data represent a mean value ± S.E.M from three independent experiments made in duplicate

Activation of adenylate cyclase at the cell membrane induces dissociation of PKA holoenzyme, translocation of PKAc to the nucleus, phosphorylation of the transcription factor CREB (cAMP response element binding protein) at Ser133 and phosphoCREB/co-activator-dependent activation of nuclear gene transcription^10,50^. Our data indicate that upon cAMP stimulation PKAc is ubiquitylated and degraded through the proteasome. By downregulating PKAc levels, CHIP may act as a control system that attenuates cAMP signaling. We tested this hypothesis by complementary approaches of gene silencing, pharmacological inhibition, and gene knock-out strategies, both in vitro and in vivo. First, we monitored the physiological consequences of CHIP downregulation on cAMP-induced phosphorylation of CREB at Ser133. Figure 6a shows that CHIP silencing increased the basal CREB phosphorylation. The effects of CHIP downregulation were dependent on increased basal PKAc activity. Thus, treating CHIP-silenced cells with the PKA inhibitor H89 reduced CREB phosphorylation to the basal values of unstimulated control cells (Fig. 6a). Similar findings were replicated by transiently expressing the K30A mutant (Supplementary Fig. 8a). To support the biochemical data, we performed in situ immunostaining analyses using anti-phosphoSer133-CREB antibodies. In control cells, FSK stimulation increased the number of phosphoCREB-positive nuclei (Fig. 6b, c**)**. In CHIP-silenced cells, the number of phosphoCREB-positive nuclei increased by several fold over the control basal value (siCNT) and was not further increased by FSK treatment. As above, H89 treatment of CHIP-silenced cells reduced phosphoCREB stainings (Fig. 6b, c).

Transcription of c-fos is mediated by direct binding of phosphoCREB to an upstream cAMP-responsive element (CRE)^51^. Given its role in CREB phosphorylation, we monitored the levels of c-fos mRNA in CHIP-depleted cells. Figure 6d shows that downregulation of CHIP enhanced basal levels of *c*-fos mRNA, reducing the fold increase induced by FSK. The effects of CHIP were abolished by pretreating the cells with H89, supporting the notion that CHIP acts as negative regulator of nuclear cAMP signaling. CHIP ubiquitinates Hsp70-bound proteins, which are actually the substrates for CHIP ubiquitylation^52^. Therefore, we evaluated the biological consequences of HSP70 inhibition on PKAc-directed CREB phosphorylation and activity. In agreement with CHIP silencing experiments, treatment with the HSP70 inhibitor (VER155008) enhanced basal levels of phosphorylated CREB (Fig. 6e, f) and of c-fos mRNA (Fig. 6g). Further, VER155008 prevented further induction of CREB phosphorylation by FSK. The contribution of PKAc ubiquitylation at K310 in the control of cAMP nuclear signaling was monitored in cells expressing the PKAc K310A mutant. As shown in Fig. 6h, i, and Supplementary Fig. 8b the K310A mutation enhanced CREB phosphorylation and c-fos transcription, mimicking the effects of CHIP downregulation or HSP70 inhibition.

### CHIP inactivation enhances neuronal cAMP signaling and LTP

**Fig. 7:**
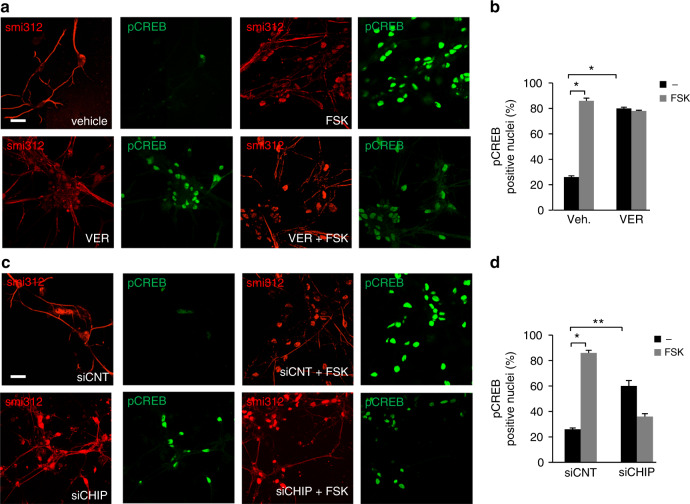
CHIP regulates cAMP signaling in neurons. **a** Primary hippocampal neurons were left untreated or stimulated with FSK (40 μM/30 min). Where indicated, cells were pretreated with VER155008 or vehicle. Formalin-fixed neurons were immunostained for pCREB and smi312, a neurofilament marker, and analyzed by confocal microscopy. Scale bar, 20 μm. **b** Quantitative analysis of the experiments shown in **a**. A mean value ± S.E.M. of three independent experiments is shown. Student’s *t* test, *p < 0.05. **c** Primary hippocampal neurons were transfected with siRNAs (control or siRNA targeting CHIP), serum deprived overnight and stimulated with FSK (40 μM/30 min). Formalin-fixed neurons were immunostained for pCREB and smi312. Scale bar, 20 μm. **d** Quantitative analysis of the experiments shown in **c**. A mean value ± S.E.M. of three independent experiments is shown. A total of 50–100 cells were scored in each set of experiments. Student’s *t* test, ^**^p < 0.01

**Fig. 8:**
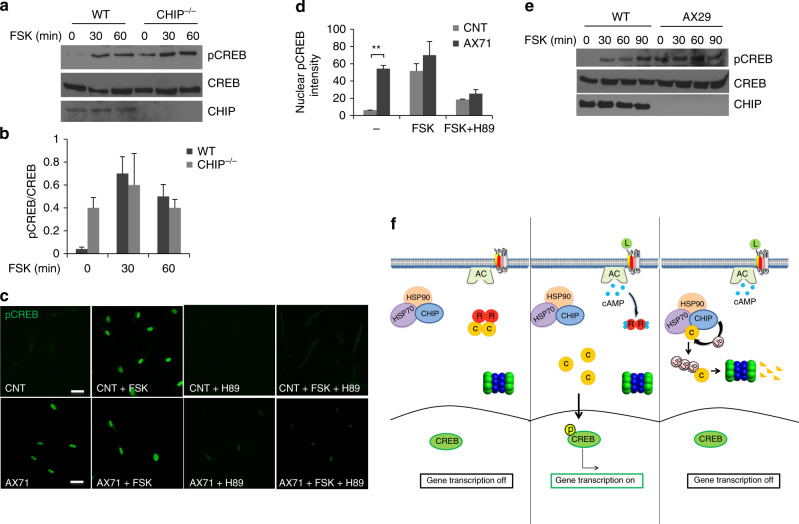
Deregulated cAMP signaling in CHIP KO mice and in SCAR16 fibroblasts. **a** Mouse embryonic fibroblasts (MEFs) isolated from control mice (WT) or from CHIP KO mice (CHIP^−/−^) were serum deprived overnight and then stimulated with FSK (40 μM). Lysates were immunoblotted for  pCREB, CREB, and CHIP. **b** Quantitative analysis of the experiments shown in **a**. A mean value ± S.E.M. of three independent experiments is shown. **c** Primary fibroblasts isolated from healthy volunteers (CNT) or from SCAR16 patient (AX71) were serum deprived overnight and stimulated with FSK (40 μM) for the indicated time points. Where indicated, cells were pretreated with H89 (10 μM). Formalin-fixed cells were immunostained for pCREB and analyzed by confocal microscopy. Scale bar, 20 μm. **d** Quantitative analysis of the experiments shown in **c**. A mean value ± S.E.M. of three independent experiments is reported. Student’s *t* test, **p < 0.01. **e** Immunoblot analysis of lysates from a different SCAR16 fibroblasts (AX29) left untreated or stimulated with FSK (40 μM) for the indicated time points using the indicated antibodies. **f** Schematic model of CHIP action on PKA signaling. Stimulation of specific GPCRs activates adenylate cyclase (AC) and increases cAMP levels, which, in turn, dissociates the PKA holoenzyme. Free, active PKAc phosphorylates (amongst other substrates) CREB at serine 133 which drives CREB-dependent gene transcription. CHIP-driven ubiquitylation and proteolysis of PKAc desensitizes receptor-cAMP mediated signal transmission

CHIP and HSP70 are abundantly expressed in mammalian brain where they play a major role in maintaining protein homeostasis and neuronal activity^34,53^. This prompted us to investigate the contribution of CHIP/HSP70 to the regulation of PKAc signaling in neurons and in intact mouse brain. First, we used primary cultures of hippocampal neurons to test the effects of HSP70 inhibition or CHIP downregulation on cAMP signaling. As shown in Fig. 7a, b, treatment with FSK in control neurons increased the number of phosphoSer133-CREB positive nuclei by three to fourfold over basal. Treatment of neurons with VER155008 markedly increased phosphoCREB-positive nuclei. A similar increase was seen in CHIP-silenced neurons (Fig. 7c, d). To further support the role of CHIP in the regulation of cAMP signaling, we monitored CREB phosphorylation in MEFs isolated from CHIP knockout mice (CHIP^−/−^). Cells were serum-deprived overnight and then stimulated with FSK. We found that deletion of CHIP increased basal CREB phosphorylation compared to control MEFs, significantly reducing the fold-induction of CREB phosphorylation by FSK treatment (Fig. 8a, b).

The data highlight that CHIP is the principal attenuating system of cAMP-PKA effectors signaling. This led us to probe the function of the CHIP system in vivo. Specifically, we investigated the electrophysiological consequences of CHIP downregulation or HSP70 inhibition on long-term potentiation (LTP) at CA1 hippocampal synapses. First, we analyzed input/output relationships by correlating slopes of evoked fEPSP with the respective population spike amplitudes. This analysis revealed that fast glutamatergic transmission is similar in control (*n* = 9 slices from 6 animals), in CHIP^−/−^ and in VER155008-treated slices (*n* = 9 slices from 8 animals) (Supplementary Fig. 9a). We then investigated the effect of VER155008 on the magnitude of cLTP. The cLTP was induced by bath application of forskolin (50 µM), the phosphodiesterase inhibitor rolipram (0.1 µM) and the G alpha i inhibitor PTX (50 µM) for 15 min, according to previous protocols^54,55^. This protocol triggered a stable cLTP in control conditions (178 ± 14% above baseline; *n* = 6). The magnitude of cLTP was further increased in by VER155008 (230 ± 14% above baseline; *p* < 0.05 vs. control LTP; *n* = 5) (Supplementary Fig. 9b). Interestingly, we obtained similar effects on cLTP using hippocampal slices isolated from CHIP^-/-^ mice (233 ± 11% above baseline; *p* < 0.05 vs. control LTP; *n* = 9) (Supplementary Fig. 9b). Taken together, our results indicate that CHIP and HSP70 activity are modulators of cAMP-dependent synaptic plasticity processes.

### Deregulated PKA signaling in SCAR16

Biallelic *STUB1* mutations that cause inactivation of CHIP have been identified in patients affected by autosomal recessive spinocerebellar ataxia-16 (SCAR16)^56,57^. SCAR16 is a rare genetic disorder characterized by truncal and limb ataxia, resulting in gait instability, mild peripheral sensory neuropathy, and cognitive defects^58,59^. Patients often present clinical signs of hypogonadism (Gordon Holmes syndrome, GHS), consistent with signaling defects and altered responses to hypothalamic hormones^58^. The phenotype of STUB1/CHIP knockout mice recapitulates most of the SCAR16 features^32^. Accordingly, we analyzed CREB phosphorylation as a readout of PKA activation in primary fibroblasts isolated from cutaneous biopsies of SCAR16 patients or from healthy volunteers. The analysis was conducted with fibroblasts isolated from two distinct SCAR16 patients, carrying inactivating CHIP mutations^56,60^. Figure 8c shows that STUB1 mutations, like CHIP downregulation, increased basal levels of phosphoCREB-positive nuclei that could not be further induced by FSK. In SCAR16 fibroblasts, inhibition of PKA activity reversed the effects of CHIP mutations on CREB phosphorylation (Fig. 8c, d). Biochemical analysis underlined the central role of CHIP in the regulation of cAMP-induced CREB phosphorylation (Fig. 8e).

Altogether, these findings highlight the existence of an UPS involved, feed-back mechanism regulating PKAc abundance. It involves the dynamically controlled participation of CHIP:kinase interactions in desensitizing cAMP-effector signaling in physiological and pathological settings in vivo.

## Discussion

Here, we report the identification of feedback regulation and phosphotransferase desensitization loop that controls the extent and duration of cAMP effector signaling based on ubiquitin-dependent proteolysis of the catalytic subunit of PKA (PKAc). Degradation of PKAc attenuates its downstream pathways. Here, we present evidence that CHIP is indeed the E3 ub-ligase responsible for ubiquitylation of activated PKAc. Interfering with CHIP expression or activity significantly affected PKAc stability and downstream signaling. Similarly, germline-inactivating mutations of CHIP, as seen in the SCAR16 syndrome, sustained cAMP signaling.

PKA plays a major role in distinct biological processes, such as metabolism, differentiation, survival, and cell growth. Fine-tuning of PKA activation is essential for cell physiology and tissue homeostasis^61^. In the course of hormone stimulation, activation of phosphodiesterases, Ser/Thr phosphatases, inhibition of adenylate cyclase, and transcriptional regulation of components of the cAMP signaling cascade all contribute to the establishment and maintenance of the refractory phase, a poststimulus period characterized by a reduced responsiveness of cells to the next cAMP wave. Inhibition of PKAc activity by PKI participates in the feed-back regulation of cAMP signaling. PKI favors the exit of free PKAc from the nuclear compartments, abrogating CREB phosphorylation and cAMP-dependent gene transcription. Together, these mechanisms constitute an important negative-feedback circuit that spatiotemporally tunes PKA activity, shaping cAMP signaling, and controlling the rate and magnitude of downstream kinase activation.

Downregulation of free PKAc levels might also contribute to the establishment of this refractory phase^17,62–64^. However, the mechanism(s) controlling the levels of PKAc in hormone-stimulated cells was principally unknown. Here, we report a control mechanism of cAMP signal attenuation based on the ubiquitin-dependent proteolysis of PKAc. Following adenylate cyclase activation, PKAc is rapidly ubiquitylated at K310. Ubiquitinated PKAc undergoes degradation through the proteasome. Decreasing PKAc levels attenuates the cAMP pathway (Fig. 8g). We showed that K310 PKAc ubiquitylation specifically affected PKAc dynamics. Indeed, MD simulations of PKAc ubiquitylated at K310, showed increased correlation among C-lobe residues, compared to free PKAc or to PKAc ubiquitylated at K286. Notably, we found a specific allosteric communication between K310 and E128, involved in ATP binding, inducing a weakening of ATP interactions. Thus, ubiquitination at K310 may contribute to signal attenuation even before proteasomal degradation. This mechanism was experimentally addressed by monitoring the kinase activity in cells expressing a mutant ubiquitin mojety lacking all the lysine residues required for ubiquitin chain elongation. Thus, mutant ubiquitin dramatically inhibited cAMP-induced activity of wild-type PKAc, but had no effects on mutant K310A.

We identified CHIP as the E3 ligase responsible of PKAc ubiquitylation. A complex, including PKAc, CHIP, and HSP90/70 is assembled in cells upon cAMP stimulation. CHIP acts as a chaperone-assisted E3 ligase primarily involved in the ubiquitylation and degradation of HSPs-bound unfolded substrates. Under stress conditions, CHIP and its co-chaperones work as the major protein quality control system^30,65^. Failed clearance of unfolded modified proteins by cytosolic chaperones-bound CHIP has been mechanistically linked to neurodegenerative disorders^53^. CHIP also controls the stability of protein kinases involved in different aspects of cell physiology^43,52,66^, suggesting that CHIP employs a dual function, as regulator of protein homeostasis and as an ON/OFF switch system for cell signaling.

Previous work identified the lysosome-targeted ARHGAP36 as a regulator of PKAc stability. Ubiquitylation at K286 induced by ARHGAP36 promotes lysosomal degradation of a restricted pool of PKAc, with, however, no major consequences on the global PKA signaling. The restricted localization of ARHGAP36 in lysosomal compartments and its highly selective expression in the pituitary gland, hypothalamus, and breast do not support a general role of ARHGAP36 in feed-back control of cAMP signaling^27^.

Our paper provides evidence, that a CHIP/HSP70 complex, in fact, controls the levels of PKAc thereby impacting on downstream signaling. Specifically, we found that CHIP ubiquitylates PKAc, promoting its proteolysis through the proteasome. In hormone-stimulated cells, K310 is the major acceptor site of ubiquitin moieties and the principal regulatory site of PKAc stability. Mutagenesis of K310 affected ubiquitylation and proteolysis of PKAc, showing significant effects on CREB phosphorylation and on nuclear c-fos transcription. This finding supports a major role of the UPS-mediated feedback regulation and desensitization of PKAc signaling. This is a general mechanism since it operates in different cell types (kidney, fibroblasts, and neurons) and also in mouse brain. Interfering with CHIP expression or activity markedly affected the amplitude and duration of PKA signaling. In vivo, deletion of CHIP or pharmacological inhibition of HSP70 activity significantly enhanced cAMP-dependent synaptic plasticity.

The role of CHIP in PKAc signaling was further supported by studies in the SCAR16 disease, a genetic syndrome caused by germline CHIP mutations. The patients present with neurological and cognitive deficits that are often coupled to hypogonadism due to altered pituitary responses to hypothalamic releasing hormones (GHS)^56^. Mice lacking the CHIP gene show a phenotype that recapitulates most of the aspects of human GHS, confirming the role of CHIP inactivation in the pathogenic features of GHS^32^. The presence of gonadothropin deficiency in GHS suggests that CHIP, beyond its major role in protein quality control system, is also involved in the regulation of the hormone-induced GPCR pathway. However, if/whether dysregulation of PKA pathway occurs in SCAR16 patients was basically unknown. Here, we provide clear evidence of dysregulation of PKA signaling in SCAR16 disease. Skin fibroblasts isolated from SCAR16 patients show an increased activation of PKA signaling that cannot be further induced by adenylyl cyclase activation. The defects of cAMP signaling in SCAR16 cells were reproduced in MEFs isolated from CHIP KO mice. In these cells, CHIP deficiency contributes to an upregulation of baseline CREB phosphorylation and c-fos transcription that was only minimally induced by cAMP agonizts. We assume that the observed disturbance of cAMP-PKA dynamics is reflected by decontrolled feedback mechanism, negatively impacting on downstream responses to GPCR ligands. These findings highlight a potential role of a deregulated CHIP•PKA pathway in the pathogenic mechanisms underlying hormonal and neurological defects present in SCAR16 patients. However, further investigation is required to better understand the mechanistic details and explain the pathological consequences.

Taken together, our findings identify the CHIP-HSP complex as an important signaling node that dynamically links cAMP oscillations with the UPS, thereby controlling the extent and duration of the cAMP-dependent PKA response. This intersection between the ubiquitin-proteasome system and second messenger controlled signaling enzymes sheds light on modes of kinase desensitization. This is relevant for physiological PKA functions related to memory consolidation, metabolism or differentiation, and should ease the design of strategies to combat PKA dysfunctions in several human disorders.

## Methods

### Cell lines

Human embryonic kidney cell line (HEK293, ATCC) and human primary fibroblasts were cultured in Dulbecco modified Eagle’s medium, containing 10% fetal bovin serum in an atmosphere of 5% CO2. Work with human SCAR16 patient cells complied with all pertinent relevant ethical regulations, and informed consent was obtained from the patients. It was approved by the Institutional Review Board of the University of Tübingen, reference number 598/2011BO1. Hippocampal primary cultures were obtained from brains of 16-day-old Wistar rat embryos, according to international guidelines for animal research and the experimental protocol approved by the Animal Care Committee of the University Federico II, Naples. Neurons were cultured at 37 °C in a humidified 5% CO_2_ atmosphere with medium replenishment after 6 days, and used after 11 days of culture in all experiments. Heterozygous Chip KO Mice were meted and plugs daily checked. A pregnant mouse was sacrificed at 12.5 d.p.c. (day postcoitum) by cervical dislocation. Both horns of uterus were removed and collected in 30 ml Dulbecco's phosphate-buffered saline (DPBS) in 10 cm dish. Individual embryos were cutted apart (including extraembryonic tissues) using blunt-sharp scissors. The embryos were dissected from the extraembryonic tissues using fine forceps and transferred to fresh dish with 20 ml cold DPBS. A single embryo was placed on Petri dish without medium for remove head and liver with fine forceps. The tissue was disrupted in Complete DMEM for Primary Cell Isolation (450 ml of DMEM, 50 ml of fetal bovine serum (FBS) (10% (v/v)), 5 ml of 200 mM l-glutamine (1/100 (v/v)), 5 ml of penicillin–streptomycin (1/100 (v/v))) by pipetting up and down 15–20 times with a pipette fitted with a 1000 µL tip to generate a single cell suspension. The MEFs cells were incubated at 37 C and 7% CO_2_. The genotype of MEFs derived from a single embryo was confirmed by PCR analysis to identify Chip Ko MEFs.

### Plasmids and transfection

Vectors encoding the Myc-tagged CHIP and the Myc-tagged Chip K30A were provided by Dr. Francesca Carlomagno (University of Naples, Italy). V5-HSP70 and pRK5-GFP-Ubiquitin-K0^67^ vectors were purchased from Addgene, HA-tagged PKA catalytic subunit type alpha and R-Luc PKA catalytic subunit wt, DNA JBI, T197A and L206R were provided by Dr. Eduard Stefan (University of Innsbruck, Austria). PKA K310A and PKA K286A were generated by site-directed mutagenesis. Human siRNA targeting coding regions of CHIP were purchased from Life Technologies: The following is the siRNA sequence, targeting human CHIP:

Sense sequence: UUACACCAACCGGGCCUUtt

Antisense sequence: CAAGGCCCGGUUGGUGUAAta

Rat siRNA targeting coding regions of CHIP were purchased from Life Technologies:

Sense sequence: GUGCCCUUCGCAUUGCUAAtt

Antisense sequence: UUAGCAAUGCGAAGGGCACta

siRNAs were transiently transfected using Lipofectamine 2000 (Invitrogen) at a final concentration of 100 pmol/ml of culture medium.

### Antibodies and chemicals

The following primary antibodies were used: Rabbit polyclonal antibodies directed against PKA alpha catalytic subunit (catalog #28315, immunoblot 1:4000), actin (catalog #sc-32251, immunoblot 1:2000) and CHIP (catalog #sc-133083, immunoblot 1:500) were bought from Santa Cruz; Myc epitope (catalog #SAB4301136, immunoblot 1:2000), V5 epitope (catalog #ABIN3181078, immunoblot 1:2000) and alpha-tubulin (catalog #T6199, immunoblot 1:8000) from Sigma; CREB (catalog #06-863, immunoblot 1:1000) and pCREB (catalog #06-519, immunoblot 1:1000, immunofluorescence dilution 1:100) from Millipore; Rluc 4410 antibodies from Millipore (catalog #MAB4410); hemaglutinin epitope HA.11 (catalog #MMS-101R, immunoblot dilution 1:1000) from Covance; mouse PKA alpha catalytic subunit (catalog #610980, immunoblot 1:2000) from BD Bioscience; rabbit HSP70 (catalog #25405-1-AP, immunoblot 1:5000) and mouse HSP90 (catalog #13171-1, immunoblot 1:5000) from Proteintech; Forskolin (catalog #66575-29-9) and 17AAG (catalog #8476) from SIGMA; VER155008 (catalog #3803) from Tocris Bioscience.

### Immunoprecipitation, immunoblot analysis, and kinase assays

Cells were washed twice with PBS and lysed in saline buffer-1% Triton-X 100 (NaCl, 150 mM; Tris-HCl, 50 mM, pH8; EDTA, 5 mM) or, for immunoprecipitation assay, in saline buffer 0.5% NP40 (50mMTris-HCl, pH 7.4, 0.15MNaCl, 100 mM EDTA,0.5% NP40) containing aprotinin (5 μg/ml), leupeptin (10 μg/ml), pepstatin (2 μg/ml), 0.5 mm PMSF, 2 mmorthovanadate, and 10 mmNaF. The lysates were cleared by centrifugation at 15,000*g* for 10 min and subjected to immunoprecipitation with the indicated antibodies. The precipiates and aliquotes of whole cell lysates (100 μg) were resolved on sodium dodecyl sulfate (SDS) polyacrylamide gel and transferred on nitrocellulose membrane for 3 h. Filters were blocked for 1 h at room temperature in Tween-20 PBS (TPBS) (PBS-Sigma, 0.1% Tween 20, pH 7.4) containing 5% nonfat dry milk. Blots were then incubated O/N with primary antibody. Blots were washed three times with TPBS buffer and then incubated for 1 h with secondary antibody (peroxidase-coupled antirabbit (GE-Healthcare)) in TPBS. Reactive signals were revealed by enhanced ECL Western Blotting analysis system (Roche). PKA enzymatic activity was analyzed in vitro on cell lysates using the PepTag Assay Kit (Promega, Madison WI, USA), following the Manufacturer’s instructions. Images of uncropped blots can be found in Supplementary Fig. 10.

### MS analysis

The C-terminus of the mature ubiquitin has the amino acid sequence KESTLHLVLRLRGG, in which the last Gly can be conjugated to lysine residues on target proteins by forming an isopeptide linkage. The tryptic digest of such modified proteins generates peptides with two N-termini carrying the characteristic reporter sequence Gly-GLy. The monoisotopic mass of this diglycine adduct is 114.04 Da. Detection of these unique mass differences or the unique peptides on lysine residues by MS in combination with the characteristic b and y ions of the fragment spectra allows for the identification of both the ubiquitination sites and the corresponding ubiquitinated proteins.

### Protein expression and purification

To obtain the prokaryotic expression vector pET42a-PKAc, the human coding region (PKAc, NP_002721.1) was PCR-amplified using oligonucleotides by insertion into the multiple cloning site of the pET42a vector (Stratagene) to get the wild-type PKAc. Expression of recombinant proteins was performed in the *E. coli* strain Rosetta pLysS (Novagen) by induction with 1 mM IPTG at 37 °C for 4 h in Luria–Bertani medium. We purified PKA GST-PKAc by affinity chromatography according to the supplier’s instructions for the Glutathione-S-transferase beads (GE Healthcare). Proteins immobilized on the beads were directly used for pull-down assays.

### GST pull-down assays

HEK293 transiently expressing both CHIP-myc and HSP70-V5 were stimulated with 30 μM Forskolin for 30 min and immediately lysed with GTPase lysis buffer (250 mM NaCl, 50 mM Tris-HCl pH 7.4, 1% Triton-X100, 1 mM EDTA pH 8.0) containing phosphatase and protease inhibitors. Cell lysates were pre-cleared with GST beads for 1 h and further incubated with the GST hybrid proteins (GST, GST-PKAc) immobilized on glutathione beads for 3 h. Resin-associated complexes were washed at least four times with GTPase lysis buffer and eluted with Laemmli sample buffer [2% SDS, 50 mM Tris·HCl pH 6.8, 0.2 mg/mL Bromophenol blue, 0.1 M DTT, 10% (vol/vol) glycerol].

### Analyses of macromolecular PKA complexes

Endogenous PKA complexes were affinity isolated from the colon cancer cell lines SW480, SW620, KM12, SKCO1, SNU175, osteosarcoma cell line U2OS, melanoma cell line A375, and human glioblastoma biopsies. Cells were grown in the appropriate media supplemented with 10% (vol/vol) FBS until they reached high cell density. Cells and tissue were homogenized (lysis buffer: 150 mM NaCl, 50 mM Tris-HCL pH7.4, 1 mM EDTA, 1% Triton, supplemented with standard protease inhibitors and phosphatase inhibitors) using a Potter S (B. Braun Biotech International) with 20 strikes. Following centrifugation (15,000×*g*, 30 min) we affinity-isolated PKA complexes using Rp-8-AHA-cAMP agarose resin (Biolog, #A012). Next, the proteomic composition of isolated PKA complexes were analyzed with LC-MS/MS on a LTQ-Orbitrap XL mass spectrometer (Thermo Fischer Scientific) coupled to a nano-flow LC system (Eksigent) and the data processing has been performed^28^.

### RNA purification and quantitative PCR analysis

Total RNA was extracted with TRIzol reagent according to the manufacturer’s protocol (Sigma). Two micrograms of the isolated RNA were reverse-transcribed with the Applyed Biosystem (Thermo fisher) kit. Real-time PCR was carried out in triplicate in 20 μl reaction volumes using the Power SYBER Green PCR Master Mix (Applied Biosystems). PCR primers for human c-*fos* mRNA are the following: forward 5′-CGGGCTTCAACGCAGACTA-3′; reverse 5′-GGTCCGTGCAGAAGTCCTG-3′. Real-time PCR reactions were carried out in a MJ Mini Personal Thermal Cycler apparatus (Bio-Rad Laboratories). Melting curves were obtained by increasing the temperature from 60 to 95 °C with a temperature transition rate of 0.5 °C s^−1^. Melting curves of final PCR products were analyzed (OpticonMonitor 3 Bio-Rad).

### Electrophysiology

Four-hundred micrometre-thick parasagittal brain slices were prepared from 2 to 3-month-old C56BL6J mice and equilibrated in artificial cerebrospinal fluid (aCSF) (in mM: 24 NaCl, 2.5 KCl, 1.25 NaH_2_PO_4_, 1.3 MgSO_4_, 2.5 CaCl_2_, 26 NaHCO_3_, 10 glucose; gassed with 95% O_2_/5% CO_2_) at room temperature for at least 1 h. Slices were transferred to a submerged recording chamber and perfused with oxygenated aCSF (2–3 ml/min) at 30–31 °C. A bipolar tungsten stimulating electrode was placed in the CA1 striatum radiatum to stimulate Schaffer collateral fibers, and extracellular field excitatory postsynaptic potentials (fEPSPs) were recorded with a glass microelectrode positioned in the stratum radiatum resistance (3–5 MΩ). Stimulus intensities were ≈40% of those that induced population spikes. For slices in which the presynaptic fiber volley was distinguishable, input–output relations were analyzed by plotting the initial slope of the fEPSP against the amplitude of the presynaptic fiber volley. LTP was induced chemically (cLTP) by a combination of forskolin/rolipram in the presence of picrotoxin (PTX)^55,68^. The effect of this protocol was expressed as mean (±SEM) percentage of baseline fEPSP amplitude measured 50 min after chemical stimulation. Statistical analysis on the average of the last 10 min of recording was evaluated by unpaired Student’s *t* test (significance was set at *p* < 0.05). Forskolin (Alomone Labs, Jerusalem, Israel) was prepared as a stock solution (50 mM, in DMSO) and stored at −80^ο^C until used at final concentration of 50 µM in the bath aCSF. Rolipram (Sigma) stock solution (0.1 mM in DMSO) was stored at −80 ^ο^C until used at final concentration of 0.1 µM. Picrotoxin (PTX Sigma, Italy) to block GABA-A-mediated activity was stored as a stock solution (5 mM, in distilled water) at 4 ^ο^C and used at 50 µM concentration. In experiments with VER155008-slices were pre-incubated for 1 h in the presence of the drug. Experiments were carried out according to international guidelines for animal research and the experimental protocol was approved by the Animal Care Committee of the University Federico II, Naples.

### PKA dynamics

The 3D structure of the hPKAc (Uniprot sequence ID P17612) was built using SWISSMODEL web server. The X-ray determined structure of PKAc in the closed form (PDB ID: 1ATP) was used as template (sequence identity of 97.2%). The global model quality estimation yielded a score of 0.98. Ubiquitin-bound PKAc was built using the X-ray structure of di-ubiquitin (PDB ID: 3NS8) as template for Ub linkage, superimposing K48 of chain B in the 3NS8 structure to K310 of hPKAc, and the bond between chain A of 3NS8 and ND of K310 was created. MD simulations were performed using the suite Amber 16 on NVIDIA Titan Xp and K20 GPUs, using the Amber99SBildn force field for the protein; ATP and phosphorylated serine and threonine parameters were taken from the Amber parameter database while parameters of Ub-linked K310 were taken from ref. ^45^. Each complex was immersed in a pre-equilibrated octahedral box of TIP3P water molecules and the system was neutralized. The final systems contained about 39000 atoms for the free PKAc and about 87,000 atoms for the K310-Ub-PKAc and K286-Ub-PKAc. Each system was minimized using energy gradient convergence criterion set to 0.01 kcal/mol Å^2^ in three steps involving: (i) hydrogen atoms in the system (5000 steps of steepest descent and 10,000 steps of conjugate gradient), (ii) hydrogen atoms, water molecules and counterions (2000 steps of steepest descent and 18,000 steps of conjugate gradient), (iii) finally the whole system (2000 steps of steepest descent and 18000 steps of conjugate gradient). Successively, the water, ions and protein side chains were thermalized in four steps of 60 ps, increasing the temperature from 50 to 298 K. During thermalization, the protein atoms were restrained using a variable restraining force: initially, a force constant of 30 kcal/mol Å^2^ was used, and subsequently decreased by increments of 5 kcal/mol Å^2^ in the next stages. Then, an additional step of 250 ps was performed in order to equilibrate the system density at constant pressure (1 bar) and temperature (298 K). Finally, an extended trajectory of 350 ns was run using a time step of 2 fs. SHAKE was used for those bonds containing hydrogen atoms in conjunction with periodic boundary conditions at constant pressure and temperature, particle mesh Ewald for the treatment of long range electrostatic interactions, and a cutoff of 10 Å for nonbonded interactions. The structural analysis was performed using in-house software and standard codes of AmberTools 17.

The ubiquitylation effects on the PKAc conformational changes have been highlighted performing the Network and communities analyses of about 1 µs-long MD trajectories of free PKAc, K310-Ub and K286-Ub PKAc using Carma ver. 0.8 plugin^69^ implemented in the VMD 1.9.2^70^. Firstly, we have defined the nodes of the network on both the PKAc and the Ubiquitin C_α_ atoms. Subsequently, in order to create the simplest networks, edges were created between pairs of nodes. In particular, edges were drawn between those nodes whose residues were within a default cut-off distance (4.5 Å) for at least 75% of our MD trajectories. Thus, the generated original network was partitioned using the Girvan-Newman algorithm into subnetworks called “communities”. Two kinds of communications can occur in each community: (i) between nodes within the same community and (ii) between nodes belonging to different communities. While, in the first case, nodes can communicate with one another relatively easily through multiple edges, in the second case, communication between different communities is reduced and generally few nodes (called “critical”) and edges are involved. This communication is defined by a metric called betweenness. This last analysis is described in detail in the Supporting Information. To understand the allosteric motions correlated to the binding of K310 to the primary active site of PKAc a weighted implementation of suboptimal paths (WISP) protocol have been applied to generate optimal and suboptimal paths. The WISP protocol calculates the primary communicating path between two residues (a source and a sink) as well as the slightly longer suboptimal paths. WISP considers each amino acid of a protein as single node (e.g., center of mass). A correlation matrix is then generated to describe the interdependence among nodes^48^. The optimal path is identified using the Dijkstra’s algorithm. To identify suboptimal paths, a recursive, bidirectional approach is employed by ignoring connections/correlations between nodes that are physically distant.

### Reporting summary

Further information on research design is available in the Nature Research Reporting Summary linked to this article.

### Supplementary information

Supplementary Information

Peer Review

Reporting Summary

Description of Additional Supplementary Files

Supplementary Movie 1

Supplementary Movie 2

### Source data

Source Data

## Data Availability

All relevant data of the present paper are available from the corresponding author on reasonable request. The source data underlying Fig. 1c, Fig. 2d–f, Fig. 3b, e, Fig. 4c, e, g, Fig. 5c, e, i, Fig. 6c, d, f, g, i, Fig. 7b, d, Fig. 8b, d, Fig. S2, Fig. S5a, b, Fig. S8b, Fig. S9a, b are provided as a Source Data file.
