# Author Correction: Feedback inhibition of cAMP effector signaling by a chaperone-assisted ubiquitin system

**DOI:** 10.1038/s41467-024-49118-y

**Published:** 2024-05-29

**Authors:** Laura Rinaldi, Rossella Delle Donne, Bruno Catalanotti, Omar Torres-Quesada, Florian Enzler, Federica Moraca, Robert Nisticò, Francesco Chiuso, Sonia Piccinin, Verena Bachmann, Herbert H. Lindner, Corrado Garbi, Antonella Scorziello, Nicola Antonino Russo, Matthis Synofzik, Ulrich Stelzl, Lucio Annunziato, Eduard Stefan, Antonio Feliciello

**Affiliations:** 1 Department of Molecular Medicine and Medical Biotechnologies, University Federico II, 80131 Naples, Italy; 2 Department of Pharmacy, University Federico II, 80131 Naples, Italy; 3 Institute of Biochemistry and Center for Molecular Biosciences, University of Innsbruck, A-, 6020 Innsbruck, Austria; 4 Department of Chemical Sciences, University Federico II, 80131 Naples, Italy; 5 European Brain Research Institute, Rita Levi-Montalcini Foundation and Department of Biology, University Tor Vergata, 00143 Rome, Italy; 6 Division of Clinical Biochemistry, Biocenter Medical University of Innsbruck, Innrain 80-82, A-6020 Innsbruck, Austria; 7 Department of Neuroscience, Reproductive and Odontostomatological Sciences, University Federico II, 80131 Naples, Italy; 8 I.R.C.S., BIOGEM, Ariano Irpino, 83031 Avellino, Italy; 9 Department of Neurodegeneration, Hertie Institute for Clinical Brain Research (HIH), University of Tübingen and German Center for Neurodegenerative Diseases (DZNE), 72076 Tübingen, Germany; 10 Institute of Pharmaceutical Sciences, University of Graz and BioTechMed-Graz, 8010 Graz, Austria; 11 IRCCS SDN, 80143 Naples, Italy

Correction to: *Nature Communications* 10.1038/s41467-019-10037-y, published online 12 June 2019

The original version of this Article contained an error in Fig. 6b. The representative image siCNT + FSK was inadvertently duplicated from the siCHIP image. The image for siCNT + FSK has been replaced with a correct image. This has now been corrected in both the PDF and HTML versions of the Article.

