# Supplementary material for: Feedback inhibition of cAMP effector signaling by a chaperone-assisted ubiquitin system
Source: Nat Commun. 2019 Jun 12;10:2572. doi: 10.1038/s41467-019-10037-y (PMC6561907; doi:10.1038/s41467-019-10037-y)
Supplement: Supplementary file 1 — Supplementary Information [file 41467_2019_10037_MOESM1_ESM.pdf]

Feedback inhibition of cAMP effector signaling by a chaperone-assisted ubiquitin system.

Rinaldi et al.

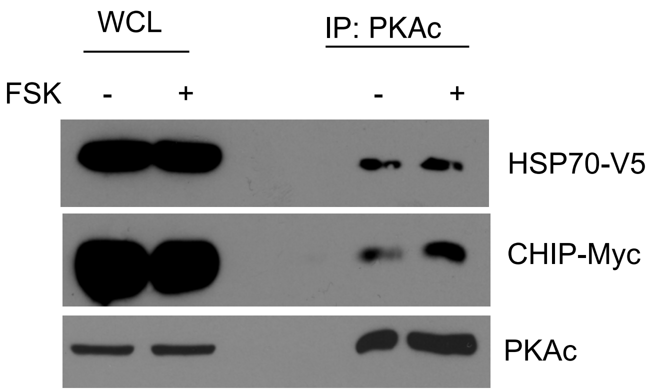

Supplementary Fig. 1. CHIP, HSP70 and PKAc form a complex in cell lysates. HEK293 cells were transiently transfected with CHIP-Myc and HSP70-V5 vectors, treated for 30 min with FSK and lysed. Lysates were immunoprecipitated with anti-PKAc antibody. The precipitates and an aliquot of lysates were immunoblotted with anti-V5, anti-PKAc and anti-myc antibodies.

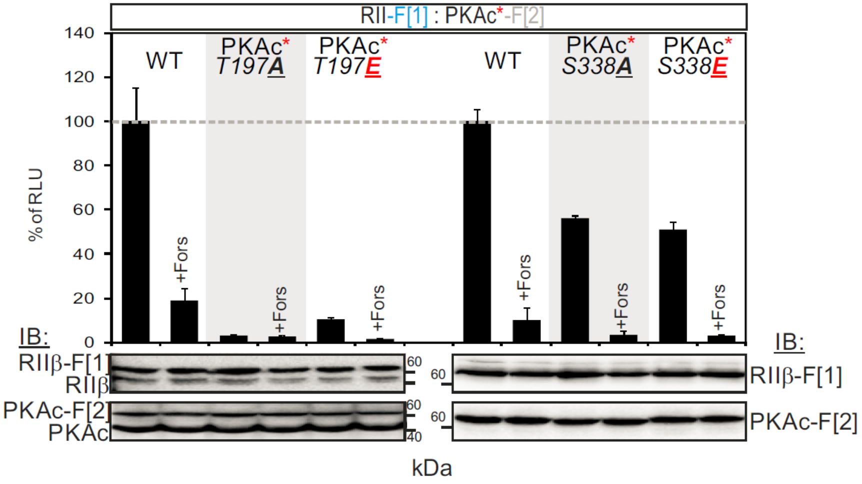

Supplementary Fig. 2. Rluc PCA analyses of RII:C PPIs. Impact of indicted mutations [*] of PKAc-F[2] on complex formation with RII-F[1]. Immunoblot indicates differences in the expression levels of untagged or tagged PKA subunits. We have used increased amounts of plasmids coding for PKAc-T197A/E-F[2] mutants to maintain similar expression levels. Student's two-tailed t-test was used to evaluate statistical significance.

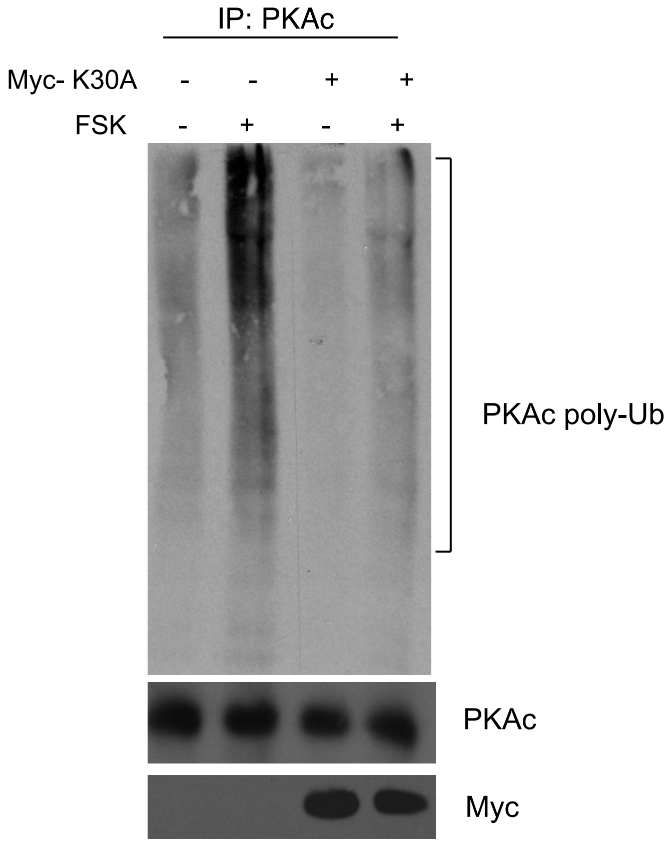

Supplementary Fig. 3. CHIP binding to HSP70 is required for cAMP-induced PKAc ubiquitylation. Lysates from cells transiently transfected with HA-ubiquitin and control vector or with CHIP-K30A vector, left antreated or stimulated with FSK, were immunoprecipitated with anti-PKAc. The precipitates were immunoblotted with anti-PKAc and anti-HA antibodies.

a

Accession #	Gene Name	Residue	Assay
P10644	PRKAR1A	K349	cAMP precip.
P31321	PRKAR1B	K349	cAMP precip.
P13861	PRKAR2A	K135	cAMP precip.
P31323	PRKAR2B	K150	cAMP precip.
P17612	PRKACA	K310	IP

b

#1	b^+	b^{2+}	Seq.	y^+	y^{2+}	#2
1	243.14517	122.07623	K-GlyGly			9
2	342.21359	171.61044	V	900.51898	450.76313	8
3	471.25619	236.13174	E	801.45056	401.22892	7
4	542.29331	271.65030	A	672.40796	336.70762	6
5	639.34608	320.17668	P	601.37084	301.18906	5
6	786.41450	393.71089	F	504.31807	252.66267	4
7	899.49857	450.25293	I	357.24965	179.12846	3
8	996.55134	498.77931	P	244.16558	122.58643	2
9			K	147.11281	74.06004	1

c

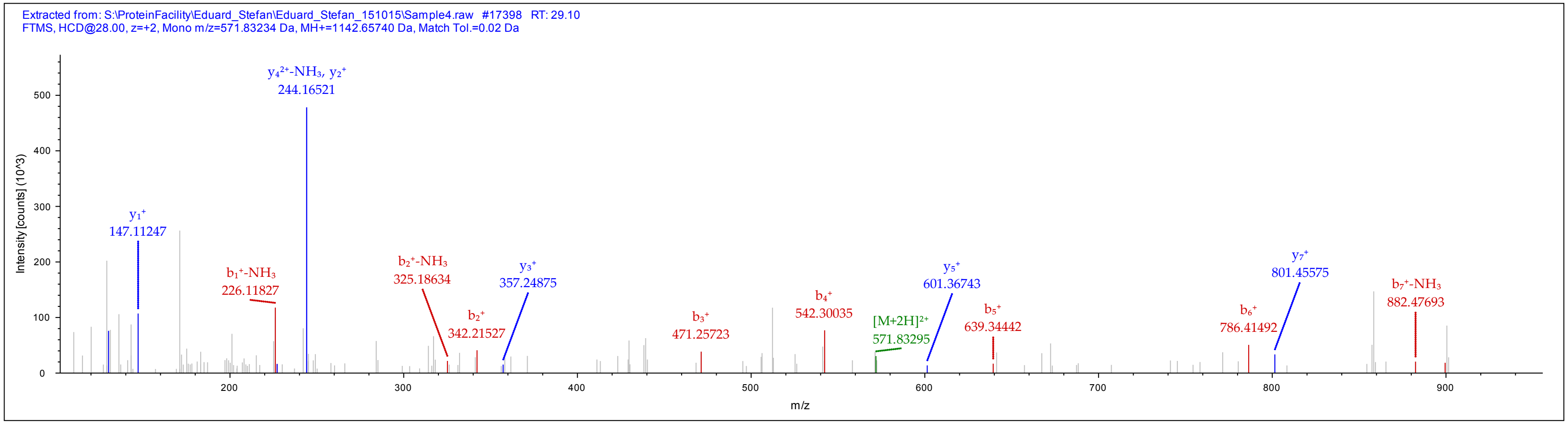

Supplementary Fig. 4. Mass spectrometry analyses of affinity isolated PKAc. (**a**) The table shows the theoretical masses of the fragment ions of the ubiquitinated peptide K(GG)VEAPFIPK. The identified fragment ions of the b-series are indicated by red numbers, the fragment ions of the y-series are indicated by blue numbers. (**b, c**)The panels show the MS/MS spectrum of the peptide K(GG)VEAPFIPK, the identified fragment ions are indicate by red and blue numbers corresponding to the masses in the table.

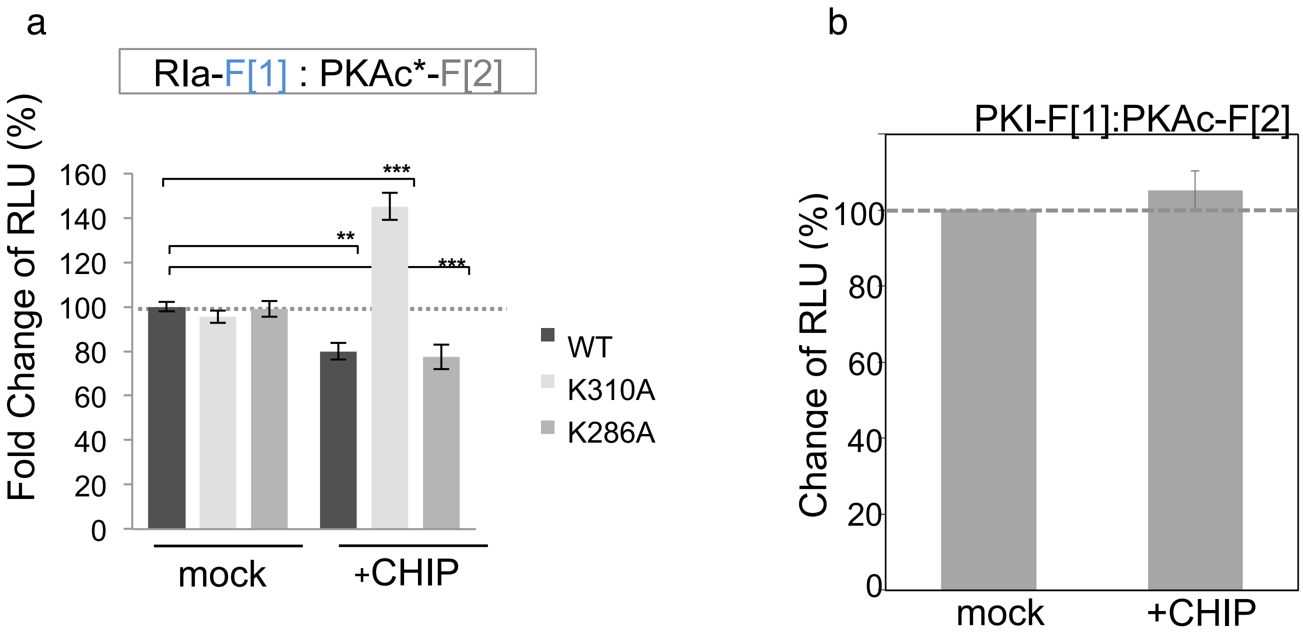

Supplementary Fig. 5. PPI analyses of RIa:C and PKI:C. PPI reporter analyses of RIa:C (**a**) and PKI:C (**b**) in cells transiently co-expressing Rluc PCA reporter constructs in the presence or absence of myc-CHIP (ratio of transfection 1:1:2). Quantification of n=5 independent experiments (mean ± SEM) are shown. Student's unpaired two-tailed T-test was used to evaluate statistical significance. **p= 0.0049; ***p<0.0001.

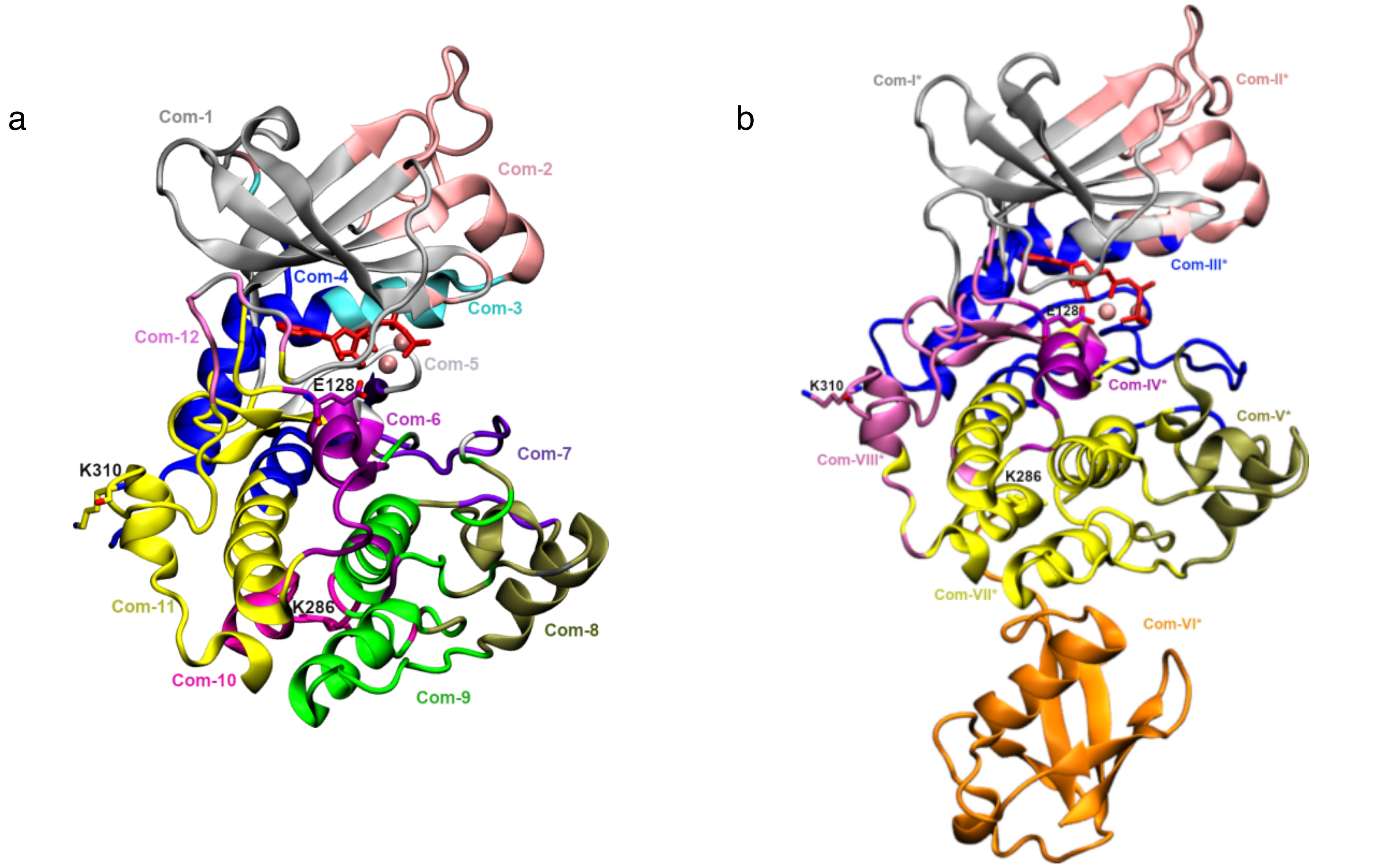

Supplementary Fig. 6. Community maps for PKA-C. **(a)** Community maps retrieved by the Dynamical Network Analysis for free-PKAc. Communities color code is detailed in Supplementary Table 2. ATP is depicted in red stick, and Mg^{++} ions are depicted in pink spheres. **(b)** Community maps retrieved by the Dynamical Network Analysis for the K286-Ub. Communities color code is detailed in Supplementary Tables 3. The Girvan-Newman algorithm splits the dynamic network of K286-Ub PKAc in 8 communities (from Com-I* to Com-VIII*).

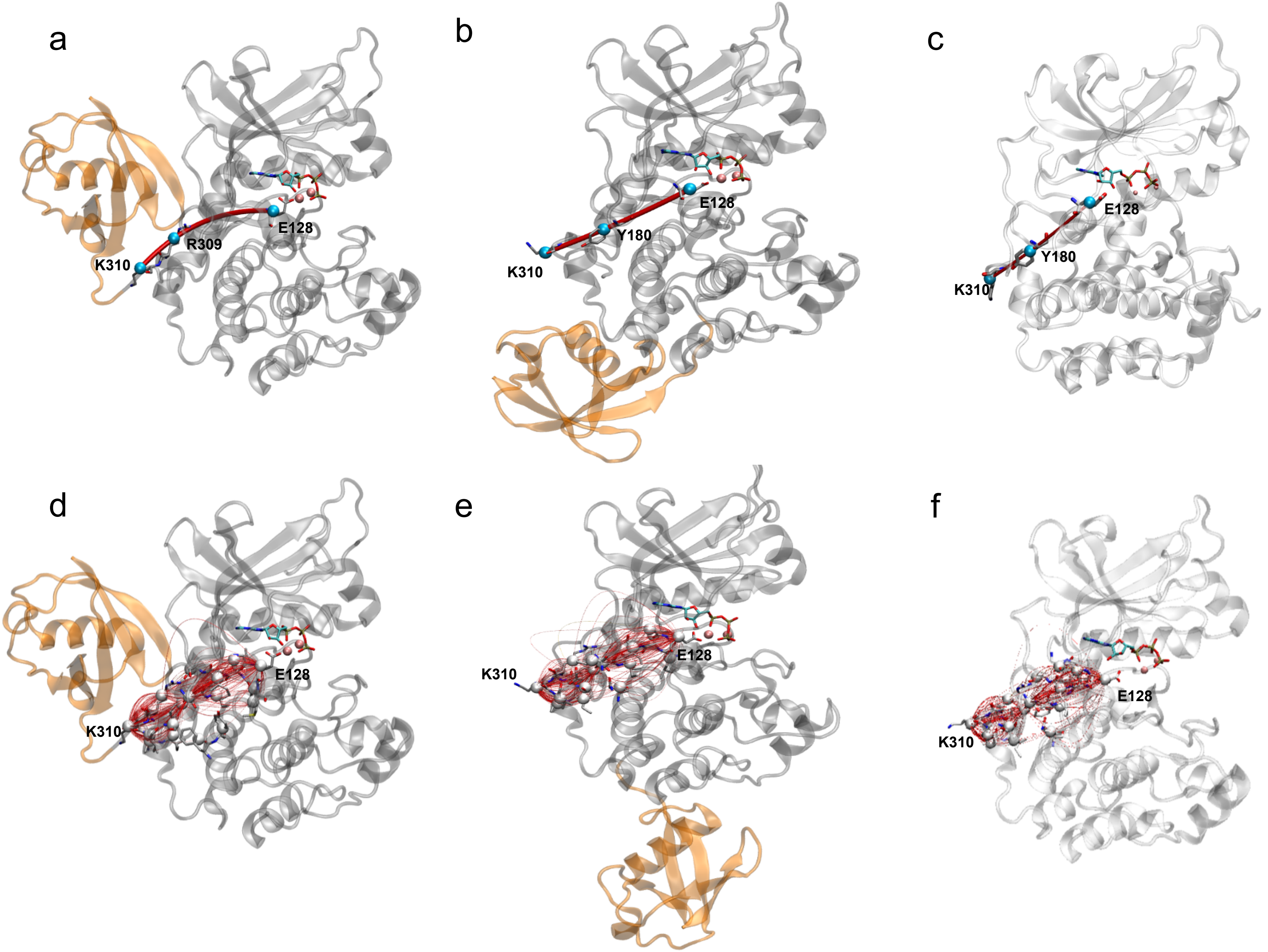
a
K310
R309
E128
b
E128
Y180
K310
c
E128
Y180
K310
d
E128
K310
e
K310
E128
f
E128
K310

Supplementary Fig. 7. Communication paths involving E128. Optimal path connecting K310 (source) and E128 (sink) during ∼1μs of MD simulation of K310-Ub PKAc **(a),** K286-Ub PKAc **(b)** and free PKAc **(c)**. 100 multiple suboptimal paths occurring between K310 (source) and E128 (sink) during ∼1μs of MD simulation of: K310-Ub PKAc **(d),** K286-Ub PKAc **(e)** and free PKAc **(f)**. The multiple suboptimal longest paths have been constructed considering a cut-off of 20 Å. PKAc and Ub are displayed as gray and orange cartoon, respectively, while ATP is represented as stick colored by atom name with cyan carbons. All the protein figures represent the average structure according to the WISP plugin protocol. White and cyan spheres represent the Center of Mass (COM) of the residues involved in the suboptimal and optimal paths, respectively. Suboptimal paths were ranked by path length, that is anticorrelated with motion correlation, so the optimal path is the one with the shortest length (i.e. most correlated motions).[2] In the K310-Ub PKAc the optimal path was K310:R309:E128 (Lenght: 1.66)**.** On the contrary, the optimal path in the K286-Ub and free PKAc form was K310:Y180:E128 (Lenght: 1.78 and 1.74, respectively), that was also the 2nd shortest optimal path in the K310-Ub PKAc (length: 1.67).

a

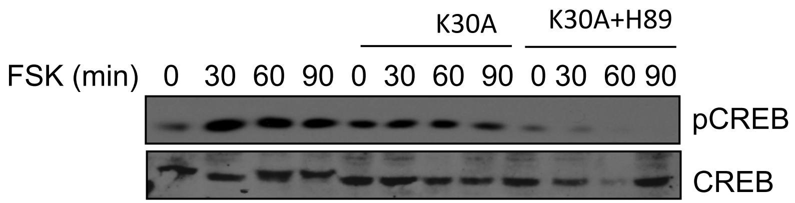

b

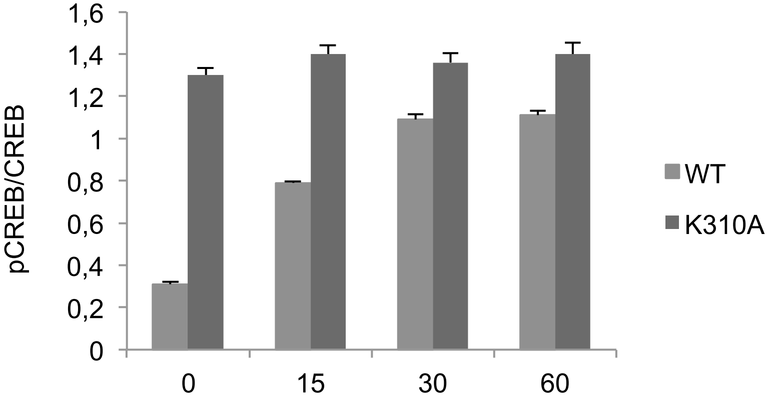

Supplementary Fig. 8. CHIP mutant affects cAMP-induced CREB phosphorylation. (**a**) Cells were transfected with control vector (CMV) or with a vector encoding for CHIP-K30A, serum deprived overnight and then stimulated with FSK. Where indicated, cells were pretreated for 1h with the PKA inhibitor H89 (10μM). Lysates were immunoblotted for phosphoSer133-CREB (pCREB) and CREB. (**b**) Quantitative analysis of two independent experiments shown in Fig 6h was performed using a Student's t-test.

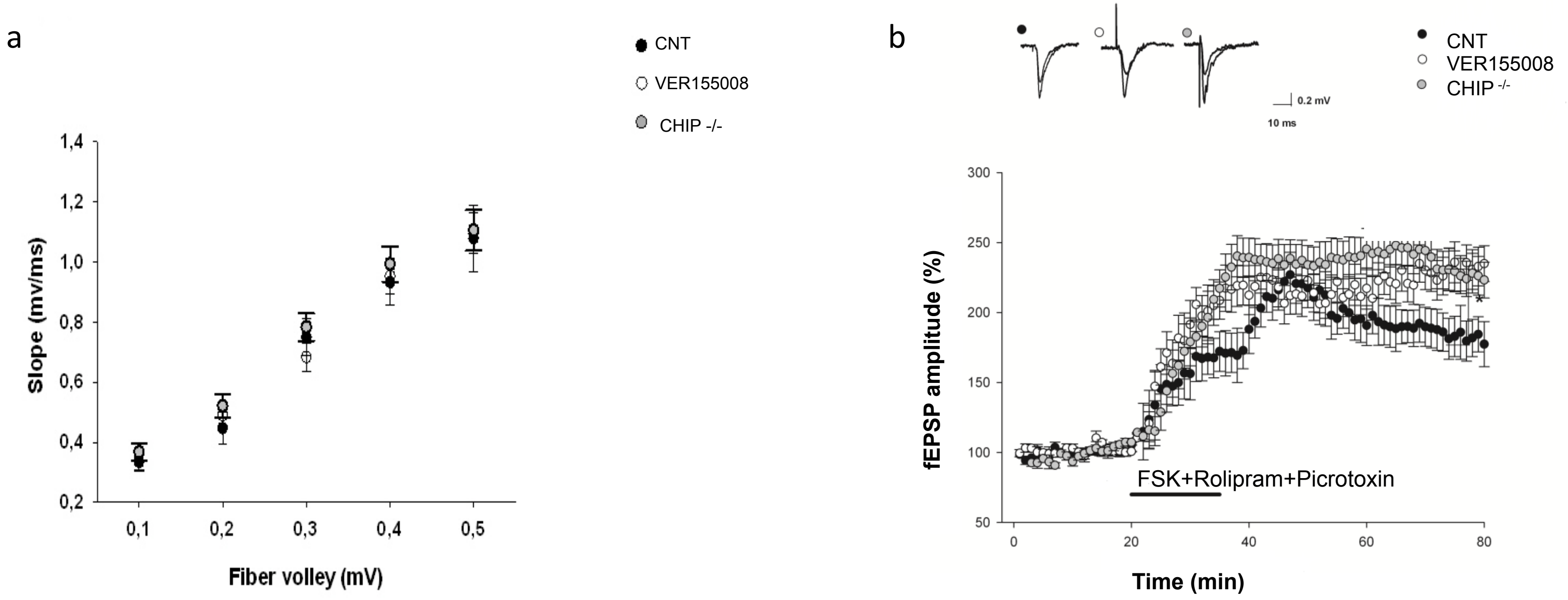

Supplementary Fig. 9. Chemical LTP in mouse hippocampal slices. **a.** Input/Output (I/O) curves for VER-treated or CHIP-/- slices vs control slices measured by plotting the field-excitatory postsynaptic potentials (fEPSP) slopes and their corresponding presynaptic fiber volley amplitudes at increasing stimulus strengths. Controls n=9 slices from 6 animals, CHIP$^{-/-}$ and in VER155008-treated slices n = 9 slices from 8 animals. **b**. Superimposed pooled data showing the normalized changes in field potential amplitude (± S.E.M.) induced by chemical protocol consisting of forskolin/rolipram application in the presence of PTX (application bar). fEPSP amplitudes were recorded and were expressed as the percentage of the baseline. Representative fEPSPs traces before and 50 min after the induction of cLTP are shown. Unpaired Student's T-test was used to evaluate statistical significance. $p<0.05$ VER155008 (n=5) vs. control LTP (n=6); $p<0.05$ CHIP$^{-/-}$ mice (n=9) vs. control LTP.

Supplementary Fig. 10

Full scans of western blots presented. The area used as data in the main figures are indicated by the red boxes.

Fig 1a

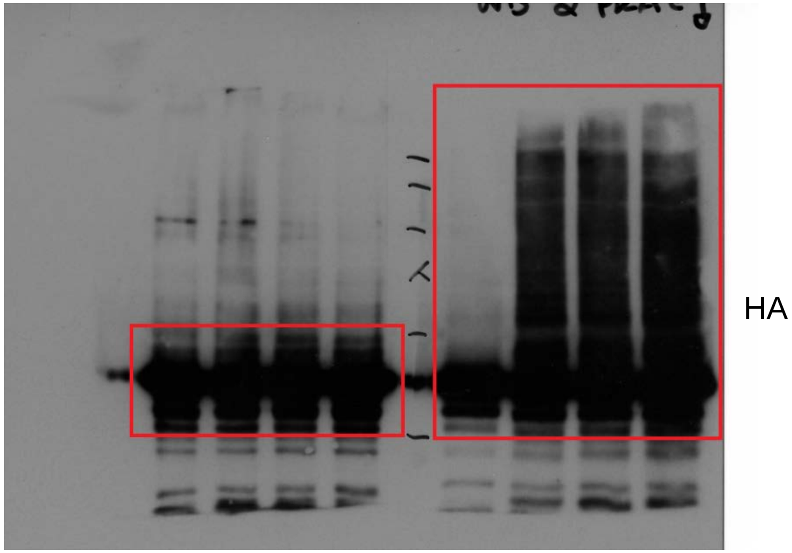

Fig 1b

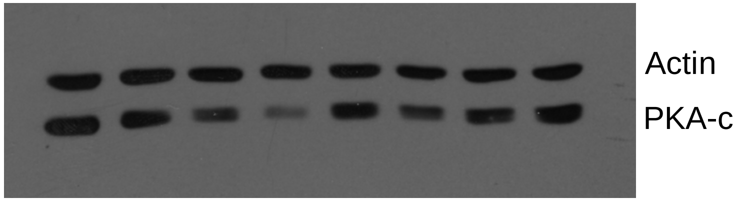

Fig 2g

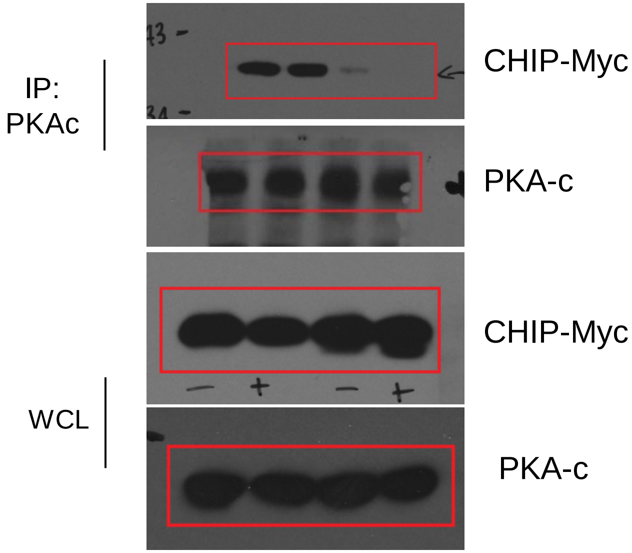

Fig 2a

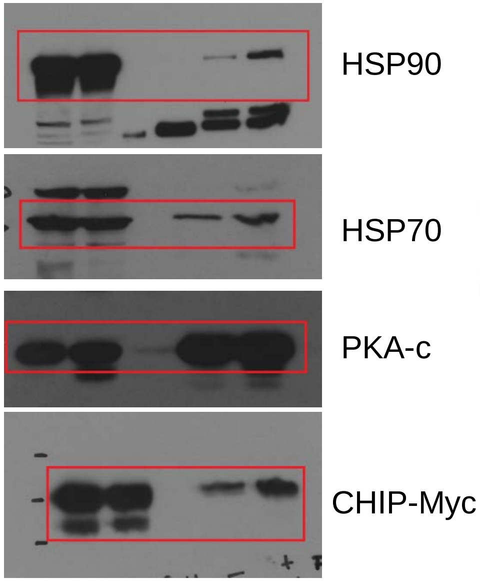

Fig 2c

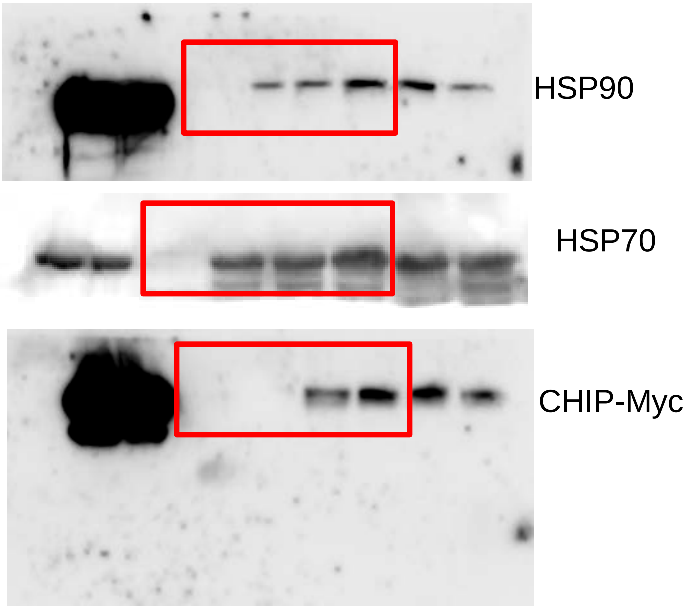

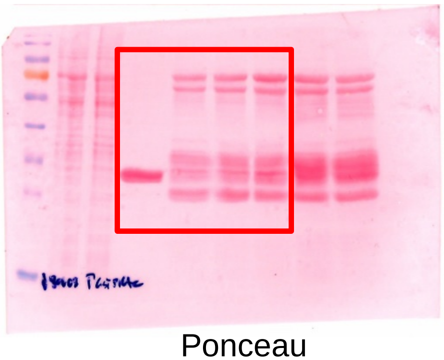

Ponceau

Fig 3a

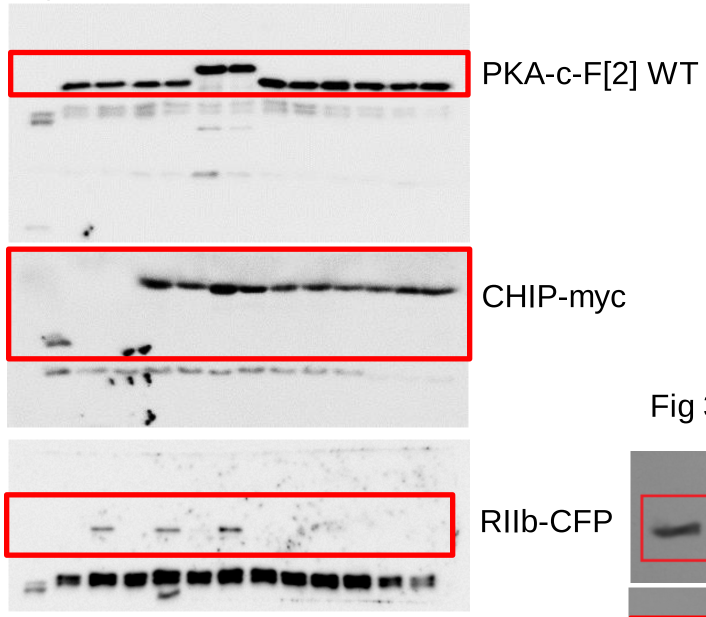

Fig 3c

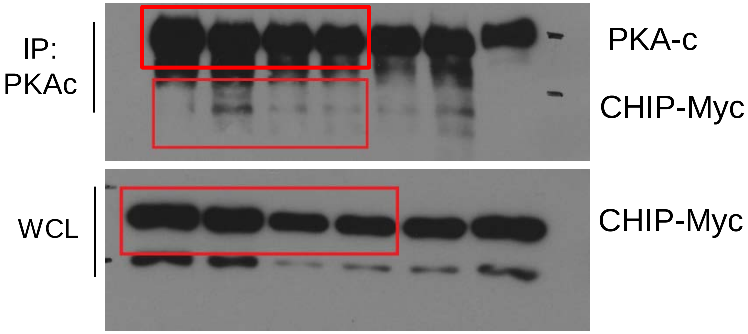

Fig 3d

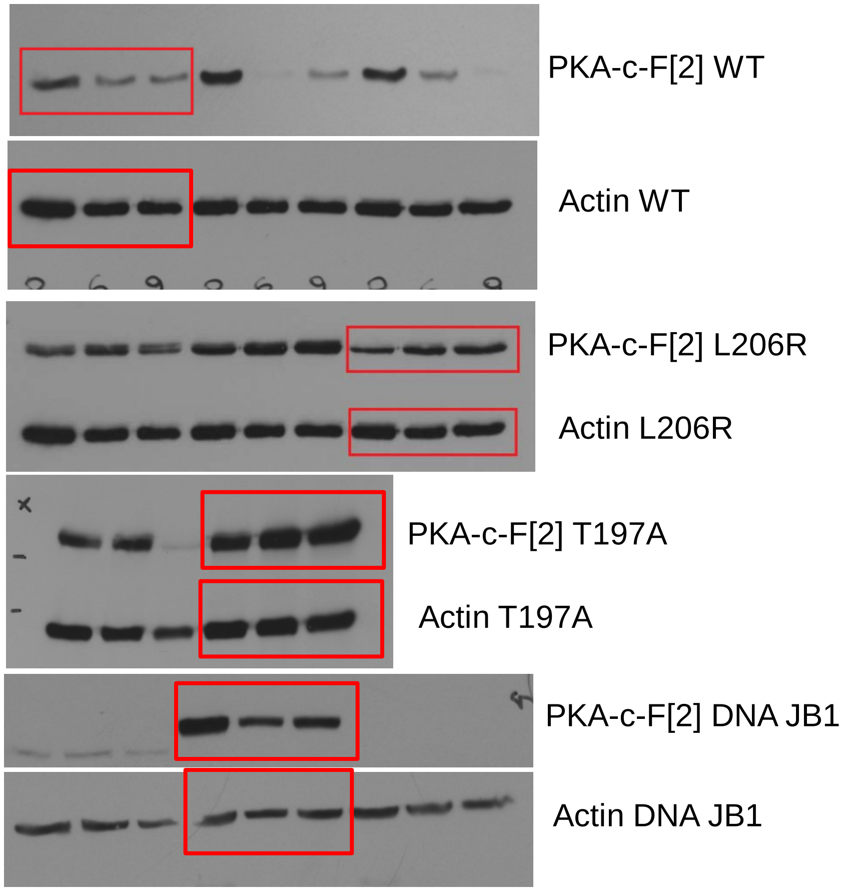

Fig 4a

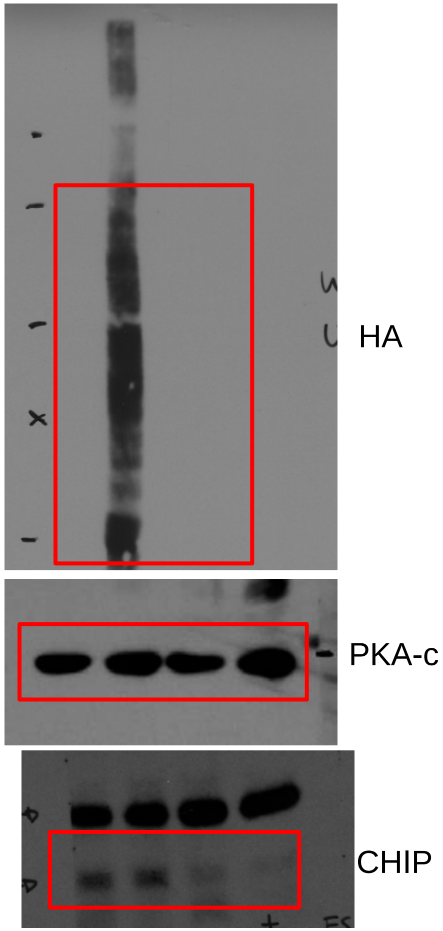

Fig 4b

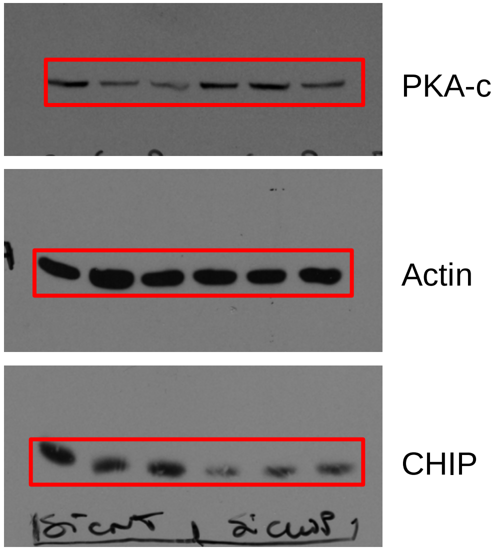

Fig 5a

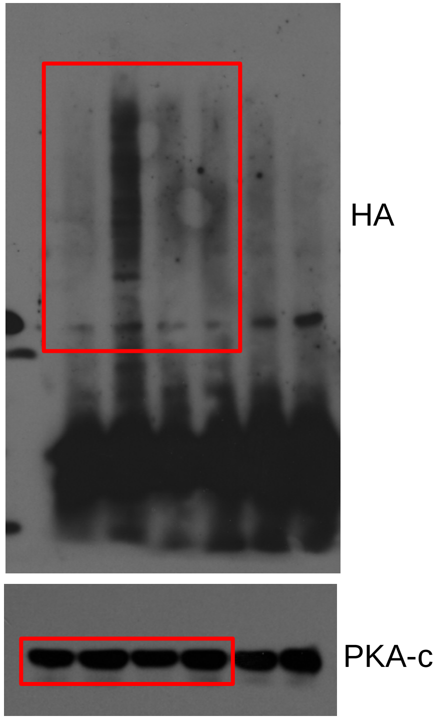

Fig 4d

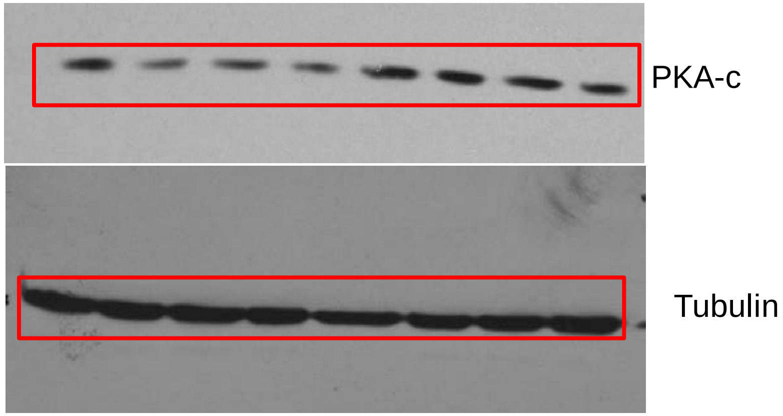

Fig 4f

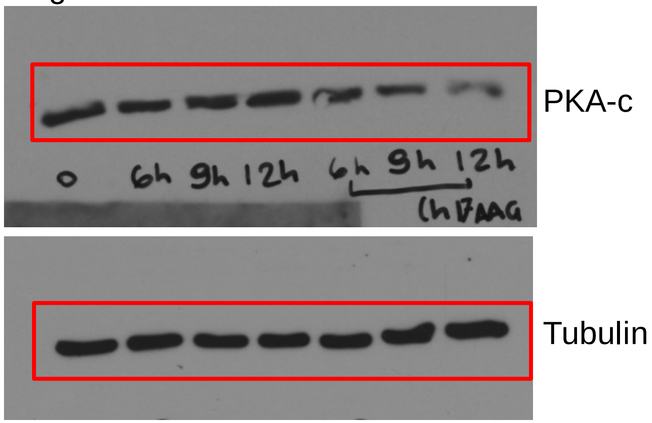

Fig 5b

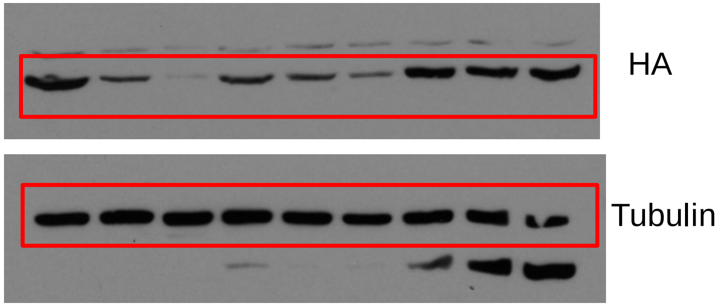

Fig 5h

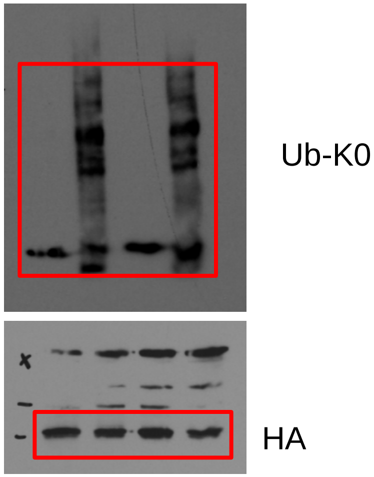

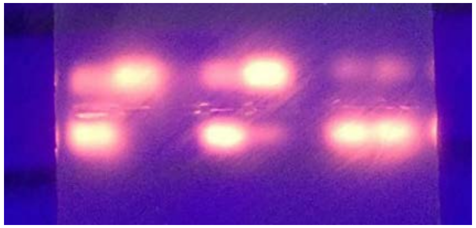

Agarose gel

Fig 6a

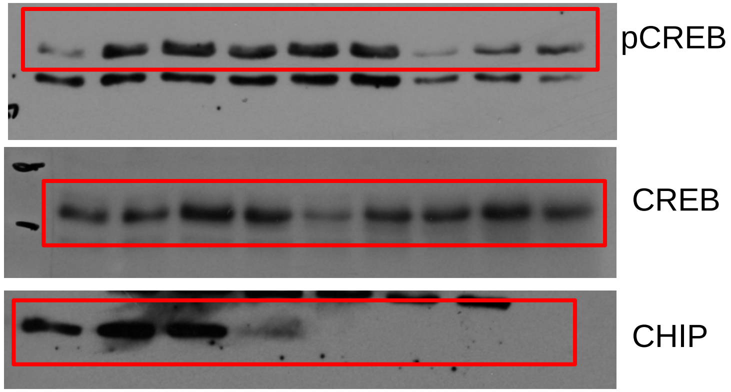

Fig 6b

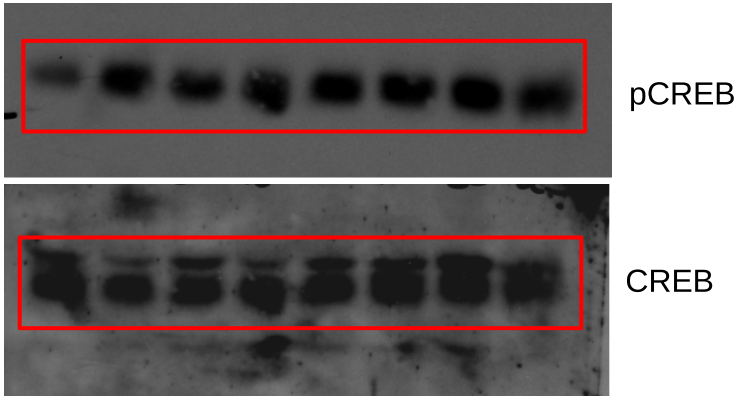

Fig 6h

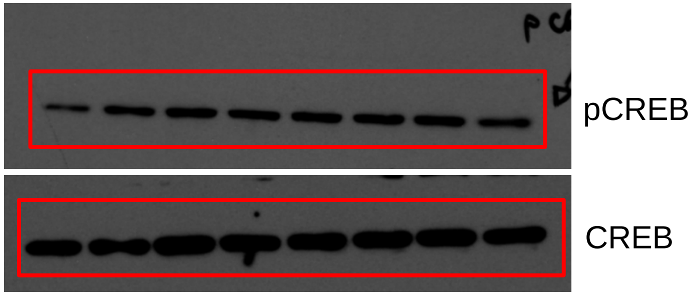

Fig 8a

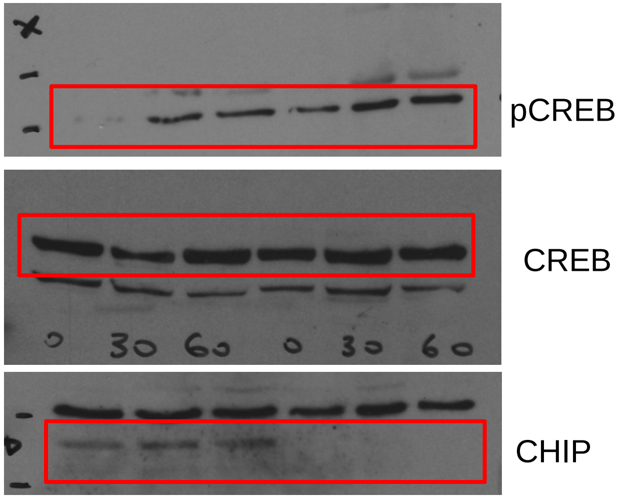

Fig 8f

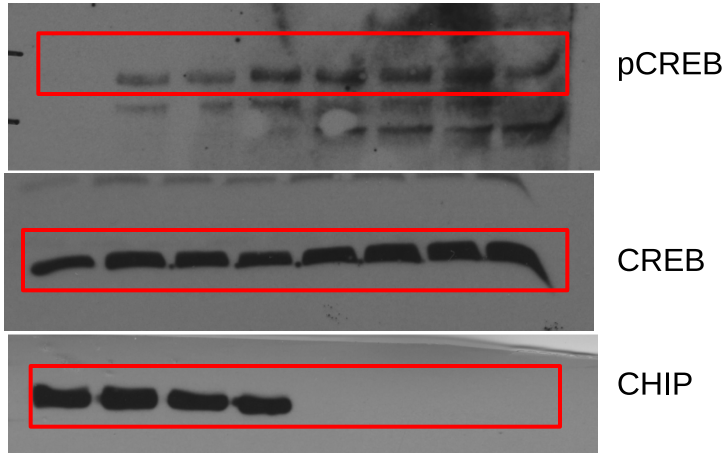

Fig S1

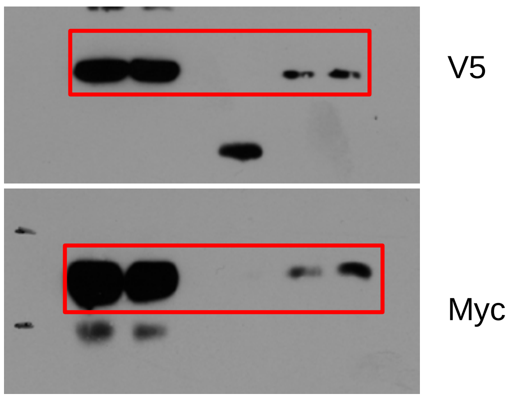

Fig S2

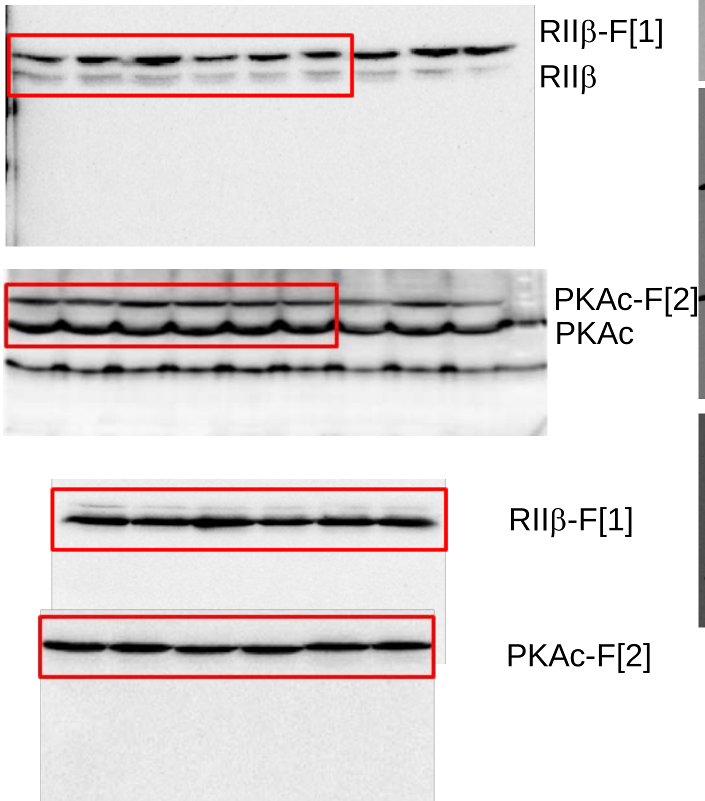

Fig S3

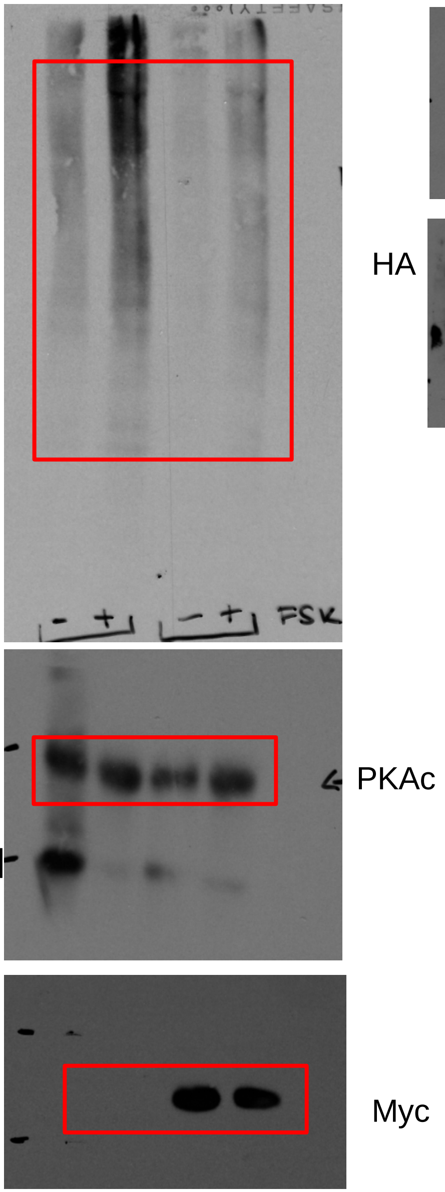

Fig S8a

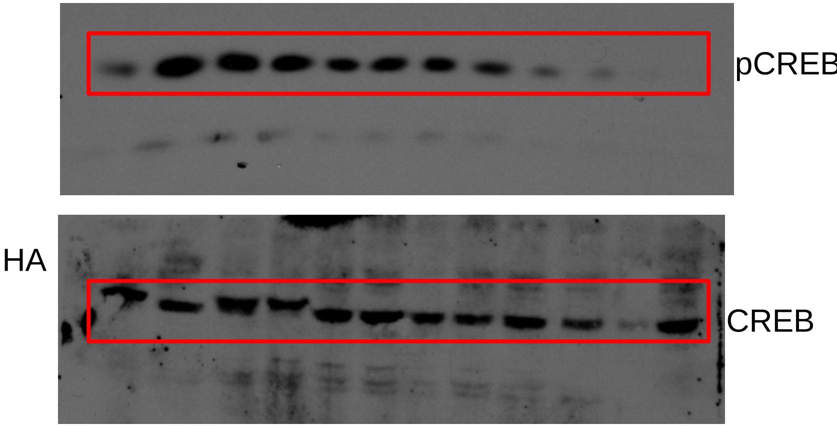

Community (K310-Ub)	N. of resid ues	Color code cartoon	McClendo n *et al.*	Function
Com-I	62	Light-gray	ComA	ATP binding
Com-II	40	Pink	ComB	Position C-helix
Com-III	36	Blue	ComC	Regulatory assembly of R-spine
Com-IV	64	Tan	*ComF* + *ComG*	Activation loop + Regulatory subunit and substrate binding
Com-V	50	Green	ComH + ComG	Protein-protein interaction site + Regulatory subunit and substrate binding
Com-VI	85	Yellow	ComE + ComD + ComC	Stabilize C-spine + Catalytic assembly of C-spine + Regulatory assembly of R-spine
Com-VII	74	Orange	*n.d*	Ubiquitin

Supplementary Table 1. Community map of K310-Ub system detected after ∼1 μs of Molecular Dynamics simulations. Each community is compared with those of the $PKAc^{ATP/2Mg^{++}}$ system.

Community (free PKAc)	N. of residues	Color code	Community (K310-Ub)	Community (K286-Ub)	McClendon *et al.*	Function
Com-1	61	Light-gray	Com-I	Com-I*	ComA	ATP binding site
Com-2	32	Pink	Com-II	Com-II*	ComB	Position C-helix
Com-3	9	Cyan	Com-II + Com-III	Com-II* + Com-III*	ComB + ComC	Position C-helix + Regulatory assembly of R-spine
Com-4	35	Blue	Com-III	Com-III*	ComC	Regulatory assembly of R-spine
Com-5	14	White	Com-III	Com-III*	ComC	Regulatory assembly of R-spine
Com-6	17	Purple	Com-VI	Com-IV*	ComD	Catalytic assembly of C-spine
Com-7	15	Violet	Com-IV	Com-III*	ComF	Activation loop
Com-8	28	Tan	Com-IV	Com-V*	ComG	Regulatory subunit and substrate binding
Com-9	49	Green	Com-V	Com-VII*	ComH	Protein-protein interaction site
Com-10	13	Magenta	Com-V	Com-VII*	ComF1	Substrate binding
Com-11	57	Yellow	Com-VI	Com-VII* + Com-VIII*	ComE + ComD	Stabilize C-spine + Catalytic assembly of C-spine
Com-12	6	Mauve	Com-I	Com-I*	ComD + ComA	Catalytic assembly of C-spine + ATP binding

Supplementary Table 2

Supplementary Table 2. Community maps of the free PKAc detected after $\sim$1 μs of Molecular Dynamics simulations. Each community is compared with the communities of the Ub-bound PKAc system and those of the PKAc$^{ATP/2Mg++}$ system.

Community (K286-Ub)	N. of residu es	Color code cartoon	McClendon *et al.*	Function
Com-I*	66	Light-gray	ComA	ATP binding
Com-II*	42	Pink	ComB	Position C-helix
Com-III*	61	Blue	ComC + ComF	Regulatory assembly of R-spine + Activation loop
Com-IV*	13	Purple	ComD	Catalytic assembly of C-spine
Com-V*	29	Tan	*ComG*	Regulatory subunit and substrate binding
Com-VI*	73	Orange	*n.d*	Ubiquitin
Com-VII*	86	Yellow	ComH + ComG + ComC	Protein-protein interaction site + Regulatory subunit and substrate binding + Regulatory assembly of R-spine
Com-VII*	37	Mauve	ComE	Stabilize C-spine

Supplementary Table 3. Community map of K286-Ub system detected after ∼1 μs of Molecular Dynamics simulations. Each community is compared with those of the PKAc$^{ATP/2Mg++}$ system .
