# Supplementary material for: Feedback inhibition of cAMP effector signaling by a chaperone-assisted ubiquitin system
Source: Nat Commun. 2019 Jun 12;10:2572. doi: 10.1038/s41467-019-10037-y (PMC6561907; doi:10.1038/s41467-019-10037-y)
Supplement: Supplementary file 3 — Reporting Summary [file 41467_2019_10037_MOESM3_ESM.pdf]

nature research

Corresponding author(s): NCOMMS-18-29786A

Reporting Summary

Nature Research wishes to improve the reproducibility of the work that we publish. This form provides structure for consistency and transparency in reporting. For further information on Nature Research policies, see Authors & Referees and the Editorial Policy Checklist.

Statistical parameters

When statistical analyses are reported, confirm that the following items are present in the relevant location (e.g. figure legend, table legend, main text, or Methods section).

n/a	Confirmed	
☐	☒	The exact sample size (n) for each experimental group/condition, given as a discrete number and unit of measurement
☐	☒	An indication of whether measurements were taken from distinct samples or whether the same sample was measured repeatedly
☐	☒	The statistical test(s) used AND whether they are one- or two-sided<br>*Only common tests should be described solely by name; describe more complex techniques in the Methods section.*
☐	☒	A description of all covariates tested
☐	☒	A description of any assumptions or corrections, such as tests of normality and adjustment for multiple comparisons
☐	☒	A full description of the statistics including central tendency (e.g. means) or other basic estimates (e.g. regression coefficient) AND variation (e.g. standard deviation) or associated estimates of uncertainty (e.g. confidence intervals)
☐	☒	For null hypothesis testing, the test statistic (e.g. F, t, r) with confidence intervals, effect sizes, degrees of freedom and P value noted<br>*Give P values as exact values whenever suitable.*
☒	☐	For Bayesian analysis, information on the choice of priors and Markov chain Monte Carlo settings
☒	☐	For hierarchical and complex designs, identification of the appropriate level for tests and full reporting of outcomes
☒	☐	Estimates of effect sizes (e.g. Cohen's d, Pearson's r), indicating how they were calculated
☐	☒	Clearly defined error bars<br>*State explicitly what error bars represent (e.g. SD, SE, CI)*

Our web collection on statistics for biologists may be useful.

Software and code

Policy information about availability of computer code

Data collection	the data were collected using Amber 15
Data analysis	R Studio software was used for T-Student's test. For structural analysis on PKAc, we used Ambertools 16; VMD 1.9.2 with Carma ver. 0.8 plugin; WISP.

For manuscripts utilizing custom algorithms or software that are central to the research but not yet described in published literature, software must be made available to editors/reviewers upon request. We strongly encourage code deposition in a community repository (e.g. GitHub). See the Nature Research guidelines for submitting code & software for further information.

Data

Policy information about availability of data

All manuscripts must include a data availability statement. This statement should provide the following information, where applicable:

- Accession codes, unique identifiers, or web links for publicly available datasets
- A list of figures that have associated raw data
- A description of any restrictions on data availability

Data on PKAc structure were from Protein Data Bank (www.rcsb.org IDs: 3NS8, 1ATP)

April 2018

Field-specific reporting

Please select the best fit for your research. If you are not sure, read the appropriate sections before making your selection.

☒ Life sciences ☐ Behavioural & social sciences ☐ Ecological, evolutionary & environmental sciences

For a reference copy of the document with all sections, see nature.com/authors/policies/ReportingSummary-flat.pdf

Life sciences study design

All studies must disclose on these points even when the disclosure is negative.

Sample size	We made no sample-size calculations. For each set of experiment sample size was extiimated to be adequate based on the magnitude and consistency of measurable differences among the groups analysed.
Data exclusions	On principle, data were only excluded for failed experiments.
Replication	Replicate experiments were successful.
Randomization	Mice analyzed were litter mates and sex-matched whenever possible and no randomization of mice was applied.
Blinding	Investigators were not blinded during experiments in vitro and in vivo.

Reporting for specific materials, systems and methods

Materials & experimental systems

n/a	Involved in the study
☐	☒ Unique biological materials
☐	☒ Antibodies
☐	☒ Eukaryotic cell lines
☒	☐ Palaeontology
☐	☒ Animals and other organisms
☐	☒ Human research participants

Methods

n/a	Involved in the study
☒	☐ ChIP-seq
☒	☐ Flow cytometry
☒	☐ MRI-based neuroimaging

Unique biological materials

Policy information about availability of materials

Obtaining unique materials	Vectors encoding for PKA and CHIP (either wt and mutants) will be available for distribution. No other unique materials were used in these studies.

Antibodies

Antibodies used	Materials and Methods Section provided with the manuscript contains information on all antibodies used in our study.
Validation	We used only commercially available, validated antibodies. For some antibodies, we confirmed the antibody specificity by competition experiments and genetic silencing.

Eukaryotic cell lines

Policy information about cell lines

Cell line source(s)	Human embryonic kidney cells were purchased from ATTC. Human primary fibroblasts were isolated from cutaneous biopsies of healthy volunteers or SCAR16 patients, following all the ethical Committee procedures. The MEFs cells were isolated from CHIP KO embryos. The genotype of MEFs derived from a single embryo was confirmed by PCR analysis to identify Chip Ko MEFs. Primary hippocampal neurons were isolated from brains of 16-d-old Wistar rat embryos, as described in Materials and Methods section

Authentication	The genotype of MEFs derived from a single embryo was confirmed by PCR analysis to identify CHIP KO MEFs. SCAR16 the Genotype of SCAR16 fibroblasts was confirmed by DNA sequencing and western blot analysis.
Mycoplasma contamination	Mycoplasm contamination was excluded using appropriate commercial kits (PCR and specific oligonucleotide primers), following the manufacturer's procedures.
Commonly misidentified lines (See ICLAC register)	No cell lines used are listed in the database of commonly misidentified cell lines.

Animals and other organisms

Policy information about studies involving animals; ARRIVE guidelines recommended for reporting animal research

Laboratory animals	Heterozygous Chip KO mice were kindly provided by Dr Cam Patterson. The genotype of CHIP KO mice was confirmed by PCR analysis to identify Chip Ko MEFs and immunoblot with specific anti-CHIP antibodies.
Wild animals	Not applicable.
Field-collected samples	The study did not involve samples collected from the field.

Human research participants

Policy information about studies involving human research participants

Population characteristics	Both subjects AX71 and AX29 suffer from a complex multisystemic autosomal-recessive ataxia caused by biallelic mutations in STUB1. AX71 is male, age 32, and AX 29 is male, age 18 years at time of fibroblast sampling.
Recruitment	Subjects were recruited and genotyped as part of a large consecutive series of early-onset ataxia patients at the Hertie Institute for Clinical Brain Research, Tübingen, Germany, by Prof Matthis Synofzik. Recruitment of subjects was approved by the IRB of Tübingen University.
