# Supplementary material for: Feedback inhibition of cAMP effector signaling by a chaperone-assisted ubiquitin system
Source: Nat Commun. 2019 Jun 12;10:2572. doi: 10.1038/s41467-019-10037-y (PMC6561907; doi:10.1038/s41467-019-10037-y)
Supplement: Supplementary file 4 — Description of Additional Supplementary Files [file 41467_2019_10037_MOESM4_ESM.docx]

**Description of Additional Supplementary Files**

File Name: Supplementary Movie 1

Description: Molecular Dynamics simulations (1ms) of K310-Ub PKAc. Ubiquitin (orange cartoon)  does not impinge the substrate-binding region (purple  cartoon) of PKAcATP/2Mg++ (white cartoon-surface), but affects the binding of ATP/2Mg++ (red sticks and pink spheres, respectively).

File Name: Supplementary Movie 2

Description: Molecular Dynamics simulations (1ms) of K286-Ub PKAc. Ubiquitin (orange cartoon)  does not impinge the substrate-binding region (purple  cartoon) of PKAcATP/2Mg++ (white cartoon-surface), and does not  affect the binding of ATP/2Mg++ (red sticks and pink spheres respectively).
