# Supplementary material for: Feedback inhibition of cAMP effector signaling by a chaperone-assisted ubiquitin system
Source: Nat Commun. 2019 Jun 12;10:2572. doi: 10.1038/s41467-019-10037-y (PMC6561907; doi:10.1038/s41467-019-10037-y)
Supplement: Supplementary file 2 — Peer Review [file 41467_2019_10037_MOESM2_ESM.pdf]

Reviewers' comments:

Reviewer #1 (Remarks to the Author):

The manuscript is well written in most parts, though a few grammatical errors are present, particularly in the first half of the manuscript. The authors have conducted studies that, to a reasonable extent, indicate that PKAc is directed for proteasomal degradation by the chaperone-mediated ubiquitination pathway involving CHIP and Hsp70. Clear experiments indicate that both CHIP and Hsp70 are necessary for the ubiquitination and experiments with K30A CHIP indicate that the interaction of Hsp70 and CHIP are also required.

The study also contains a molecular dynamics study that is a bit of a non-sequitur. It is not clear why the authors proceeded to conduct molecular dynamics studies of K310-Ub-PKAc as ubiquitination at K310 promotes proteasomal degradation of PKAc. The argument that ubiquitinaton at K310 affects the dynamics of E128 and that this exhibits a significant or observable effect is not placed on solid footing within the current manuscript. The modeling of K310-Ub-PKAc seems to neglect the fact that processive ubiquitination to polyubiquitinate PKAc would likely result in rapid proteasomal degradation of PKAc. The authors even state that "Following adenylate cyclase activation, PKAc is rapidly ubiquitylated at K310. Ubiquitinated PKAc undergoes degradation through the proteasome." The ability for data on the K310A PKAc to lead to direct claims of the contribution of K310-Ub-PKAc is unclear and not well communicated. Prior to making the claim that mono-ubiquitinated PKAc can attenuate PKAc-mediated signaling when ubiquitinated at K310 but not K286, at a minimum, the authors should produce mono-ubiquitinated PKAc-K310Ub and PKAc-K286Ub and measure PKAc signaling. Utilizing K0 ubiquitin would provide a route toward producing these intermediates.

In addition to these comments, the authors should consider the following:
1. Western blot in figure 1a is of insufficient quality to sate that PKAc-Ub(n) is observed.
2. The final sentence in the legend for Figure 3 "A mean value ± S.E.M. if it is SEM it
472 is by no means significant!! of three independent experiments is reported." should be reworded.
3. It is surprising that for such low molecular weight protein, PKAc, that for PKAc-Ub(n) a ladder of ubiquitinated PKAc is not observed.
4. It would be helpful to see the mass spectrometry data used to identify the ubiqutintation site on PKAc.

Overall this is a promising study with a range of techniques. The conclusions are interesting and will be meaningful for the field. The manuscript, in current form, could be greatly enhanced by (1) more fully connecting the molecular dynamics results to the rest of the study through biochemical experiments, (2) correcting grammar within the manuscript, and (3) improving the quality of the western blot data.

Reviewer #2 (Remarks to the Author):

This manuscript identifies a novel ubiquitin/proteasomal pathway that degrades the free C subunit of PKA. It has been known for decades that PKA-C decreases during chronic stimulation of PKA and the presumption was that proteolysis was involved. A recent paper demonstrated that ARHGAP36 promoted ubiquitylation of PKA-C and degradation by a lysosomal mechanism. This manuscript describes an alternative pathway involving the chaperones Hsp70/90 and the E3 ligase, CHIP, and demonstrates that this pathway is probably responsible for the majority of PKA-C degradation after dissociation from the holoenzyme by cAMP. This is a major contribution to the PKA field and has potential significance in terms of human disease linked to mutations in CHIP as seen in spinocerebellar ataxia-16 patients.

Minor comments and suggestions:

1. One of the earliest references describing the degradation of PKA-C is Hemmings, BA, (1986) FEBS Letters 196:126 and I suggest referencing it.
2. Fig. 4f and g are confusing. There is no forskolin in this experiment but it is indicated in the quantitation in 4g. Should add hours (h) to 4f and indicate treatment (inhibitor) or something.
3. Fig. 5: line 498 indicates ERK2 but it says tubulin on figure and actin in 5d.
4. Fig. 6: units in 6g and 6i do not match or make sense
5. line 259: reference to Supplementary Fig. 4 should be Suppl Fig. 5. Could not find how long H89 treatment was for and the 10mm should be 10uM.
6. line 277: authors say that VER155008 blocks pCREB with FSK or iso but I didn't see an experiment like this with iso.
7. Fig. 6: changes in pCREB in 6h should be quantitated
8. The statement (line 312) that CHIP and HSP70 are essential elements for cAMP-dependent long-term memory processes is overkill since there is no behavior in this manuscript and CHIP and HSP70 are involved in much more than just PKA signaling. Chemically induced LTP in the CA1-CA3 pathway does not infer long term memory.
9. line 305: Suppl Fig. 5 should be Suppl Fig. 6
10. The section using molecular dynamics to attempt to show an effect of K310 ubiquitin on C subunit function could be more concise. It is speculation and the authors have no biochemical experiment to test the hypothesis.
11. line 390: authors state that mutagenesis of K310 but not K286 had effects on CREB phosphorylation and c-fos. I did not see this data for K286 anywhere.
12. line 397: The authors state that deletion of CHIP or pharmacological inhibition of HSP70 enhanced cAMP-dependent synaptic plasticity and long-term memory. Should this be referenced? The data in Fig. 8c is not obviously significant (no stats given) and would be viewed as chemical LTP but not synaptic LTP induced by neuronal firing.
13. It is important to remind the readers that CHIP is generally thought of as a quality control pathway to protein degradation and mutations in the pathway in SCAR16 patients are likely to lead to buildup of unfolded proteins and subsequent pathology.

Reviewer #3 (Remarks to the Author):

The work by Rinaldi et al. is a great one describing a new mechanism of controlling PKAc activity and the protein mediators of it (CHIP, ubiquitylation of PKAc at lysine 310). Overall, the work is well done and the data convincing; the linkage to human disease (SCA16) is also notable. I have some major and minor edits to consider for any further work:

1. The R2b data are shown; have the authors done work with R1a? it is the main mediator of cAMP activity. R2b became prominent with the L206R work but R1a is really the most important PKA R subunit in terms of immediate cAMP effects. Some work with R1a would strengthen the work tremendously.
2. Additional demonstration of CHIP+PKAc interaction in other cells/tissues beyond what is shown here would be welcome: bone cells? adrenal?
3. How does the ubiquitylation of PKAc at lysine 310 affect interaction with PKI? it should be explored

Other minor comments:

1. In genetics, these ataxias are abbreviated as SCAs (not SCAR)
2. The LTP work is somewhat weak and I wonder whether it should remain here, especially in light of the additional work suggested above.

Reviewer #1 (Remarks to the Author):
The manuscript is well written in most parts, though a few grammatical errors are present, particularly in the first half of the manuscript. The authors have conducted studies that, to a reasonable extent, indicate that PKAc is directed for proteasomal degradation by the chaperone-mediated ubiquitination pathway involving CHIP and Hsp70. Clear experiments indicate that both CHIP and Hsp70 are necessary for the ubiquitination and experiments with K30A CHIP indicate that the interaction of Hsp70 and CHIP are also required. The study also contains a molecular dynamics study that is a bit of a non-sequitur. It is not clear why the authors proceeded to conduct molecular dynamics studies of K310-Ub-PKAc as ubiquitination at K310 promotes proteasomal degradation of PKAc. The argument that ubiquitinaton at K310 affects the dynamics of E128 and that this exhibits a significant or observable effect is not placed on solid footing within the current manuscript. The modeling of K310-Ub-PKAc seems to neglect the fact that processive ubiquitination to polyubiquitinate PKAc would likely result in rapid proteasomal degradation of PKAc. The authors even state that "Following adenylate cyclase activation, PKAc is rapidly ubiquitylated at K310. Ubiquitinated PKAc undergoes degradation through the proteasome." The ability for data on the K310A PKAc to lead to direct claims of the contribution of K310-Ub-PKAc is unclear and not well communicated. Prior to making the claim that mono-ubiquitinated PKAc can attenuate PKAc-mediated signaling when ubiquitinated at K310 but not K286, at a minimum, the authors should produce mono-ubiquitinated PKAc-K310Ub and PKAc-K286Ub and measure PKAc signaling. Utilizing K0 ubiquitin would provide a route toward producing these intermediates.

Author Response (AR): Many thanks to the Reviewer in finding our manuscript well written, promising, interesting and meaningful for the field. We have addressed the three major concerns raised by the Reviewer #1: First, we integrate data which support the molecular dynamics predictions; second, we streamlined the manuscript; third, we improved the quality of the blots.

As for the molecular dynamics, as suggested by the Reviewer, we have now analyzed the role of mono-ubiquitylation on PKAc activity by performing kinase assays on lysates isolated from cells expressing Ub-K0 mutant. This mutant lacks the lysine residues at positions 6, 11, 27, 29, 33, 48, and 63 that are critical for ubiquitin chain elongation. The data reported in the **new Figures 5h, 5i** show that co-expression of Ub-K0 significantly inhibited PKAc activity. In contrast, the activity of PKAc mutant carrying substitution of K310 to Ala cannot be inhibited by Ub-K0. These new findings, now, better support the molecular dynamics model showing that monoubiquitylation of PKAc at K310, indeed, affects the accessibility of the ATP binding pocket and hence the catalytic activity.

In addition, we clarified and addressed the following points.

1. Western blot in figure 1a is of insufficient quality to state that PKAc-Ub(n) is observed.

AR: As suggested, we have replaced the panels of Fig.1A with a new data showing high quality blots (**new Fig. 1a**).

2. The final sentence in the legend for Figure 3 "A mean value ± S.E.M. if it is SEM it

is by no means significant!! of three independent experiments is reported." should be reworded.

AR: We apologize for the confusion. We have made the appropriate correction to the text.

3. It is surprising that for such low molecular weight protein, PKAc, that for PKAc-Ub(n) a ladder of ubiquitinated PKAc is not observed.

AR: In the **new Fig. 1a**, the poly-ubiquitylation ladder is now evident.

4. It would be helpful to see the mass spectrometry data used to identify the ubiqutintation site on PKAc.

AR: As suggested, we have now included the mass spectrometry data in the **new Supplementary Fig.4b, 4c.**

Overall this is a promising study with a range of techniques. The conclusions are interesting and will be meaningful for the field. The manuscript, in current form, could be greatly enhanced by (1) more fully connecting the molecular dynamics results to the rest of the study through biochemical experiments, (2) correcting grammar within the manuscript, and (3) improving the quality of the western blot data.

AR: Thank you for your support. Besides the streamlining of the manuscript, we have integrated new figures and new text (highlighted in blue).

Reviewer #2 (Remarks to the Author):
This manuscript identifies a novel ubiquitin/proteasomal pathway that degrades the free C subunit of PKA. It has been known for decades that PKA-C decreases during chronic stimulation of PKA and the presumption was that proteolysis was involved. A recent paper demonstrated that ARHGAP36 promoted ubiquitylation of PKA-C and degradation by a lysosomal mechanism. This manuscript describes an alternative pathway involving the chaperones Hsp70/90 and the E3 ligase, CHIP, and demonstrates that this pathway is probably responsible for the majority of PKA-C degradation after dissociation from the holoenzyme by cAMP. This is a major contribution to the PKA field and has potential significance in terms of human disease linked to mutations in CHIP as seen in spinocerebellar ataxia-16 patients.

AR. Many thanks to the reviewer for considering our manuscript as a major contribution to the PKA field with potential significance in terms of human disease linked to mutations in CHIP as seen in SCAR16 patients.

Minor comments and suggestions:

1. One of the earliest references describing the degradation of PKA-C is Hemmings, BA, (1986) FEBS Letters 196:126 and I suggest referencing it.

AR: We have now integrated the reference mentioned above (**please, see line 75, and new ref. 22**) .

2. Fig. 4f and g are confusing. There is no forskolin in this experiment but it is indicated in the quantitation in 4g. Should add hours (h) to 4f and indicate treatment (inhibitor) or something.

AR: We apologize for the confusion. We have corrected the labels to the **Fig.4f, 4g**, accordingly.

3. Fig. 5: line 498 indicates ERK2 but it says tubulin on figure and actin in 5d.

AR: Corrected.

4. Fig. 6: units in 6g and 6i do not match or make sense.

AR: Corrected (**please, see new Fig.6g, 6i**).

5. line 259: reference to Supplementary Fig. 4 should be Suppl Fig. 5. Could not find how long H89 treatment was for and the 10mm should be 10uM.

AR: We apologize for the confusion. We have corrected the reference to **new Supplementary Fig. 8**. We have also included details in the main text on the time and dose of H89 treatment (line 560-561).

6. line 277: authors say that VER155008 blocks pCREB with FSK or iso but I didn't see an experiment like this with iso.

AR: Corrected.

7. Fig. 6: changes in pCREB in 6h should be quantitated.

AR: We have now included the quantitation of pCREB data in the **new Supplementary Fig. 8b.**

8. The statement (line 312) that CHIP and HSP70 are essential elements for cAMP-dependent long-term memory processes is overkill since there is no behavior in this manuscript and CHIP and HSP70 are involved in much more than just PKA signaling. Chemically induced LTP in the CA1-CA3 pathway does not infer long term memory.

AR: The referee is correct. We have now modified “long term memory” to “synaptic plasticity” (**please, see line 346**)

9. line 305: Suppl Fig. 5 should be Suppl Fig. 6

AR: Corrected.

10. The section using molecular dynamics to attempt to show an effect of K310 ubiquitin on C subunit function could be more concise. It is speculation and the authors have no biochemical

experiment to test the hypothesis.

AR: As suggested by the Reviewer, we tested the role of mono-ubiquitylation on PKAc activity by performing in vitro kinase assays on lysates isolated from cells expressing Ub-K0 mutant. This mutant lacks the lysine residues at positions 6, 11, 27, 29, 33, 48, and 63 that are critical for ubiquitin chain elongation. The data reported in the **new Fig. 5h, 5i** show that co-expression of Ub-K0 inhibits PKAc activity. In contrast, the activity of PKAc mutant carrying subtitution of K310 to ala cannot be inhibited by Ub-K0. These new findings, now, better support the molecular dynamics modeling showing that mono-ubiquitylation of PKAc at K310, indeed, affects the accessibility to the ATP binding pocket and the kinase activity.

11. line 390: authors state that mutagenesis of K310 but not K286 had effects on CREB phosphorylation and c-fos. I did not see this data for K286 anywhere.

AR: We apologize for the confusion. We corrected the text, accordingly (**please, see line 428**).

12. line 397: The authors state that deletion of CHIP or pharmacological inhibition of HSP70 enhanced cAMP-dependent synaptic plasticity and long-term memory. Should this be referenced? The data in Fig. 8c is not obviously significant (no stats given) and would be viewed as chemical LTP but not synaptic LTP induced by neuronal firing.

AR: We have inserted the statistical analysis as requested by the Reviewer (**please, see lines 343 and 345**).

13. It is important to remind the readers that CHIP is generally thought of as a quality control pathway to protein degradation and mutations in the pathway in SCAR16 patients are likely to lead to buildup of unfolded proteins and subsequent pathology.

AR: We have now appropriately addressed this point in the text (**please, see line 443**)

Reviewer #3 (Remarks to the Author):

The work by Rinaldi et al. is a great one describing a new mechanism of controlling PKAc activity and the protein mediators of it (CHIP, ubiquitylation of PKAc at lysine 310). Overall, the work is well done and the data convincing; the linkage to human disease (SCA16) is also notable.

AR: Many thanks to the Reviewer in finding our manuscript a great one, well done and the data convincing. We have addressed all the points raised by all three Reviewers and revised the manuscript accordingly.

I have some major and minor edits to consider for any further work:

1. The R2b data are shown; have the authors done work with R1a? it is the main mediator of

cAMP activity. R2b became prominent with the L206R work but R1a is really the most important PKA R subunit in terms of immediate cAMP effects. Some work with R1a would strengthen the work tremendously.

AR: We agree with Reviewer #3. RIa is indeed the key compensatory R subunit in this kinase family which has been actually confirmed by gene knockouts of the three other regulatory subunits in mice. We integrate a relevant citation (**new ref. 7, line 64**).
To show that RIa:PKAc complexes are affected as well by CHIP activities we used the PPI reporter system to show clear evidence that CHIP affects not just RII:C but also the turnover of RIa:C complexes. Mutation of K310 stabilizes both complexes in the presence of CHIP (**please, see Fig. 5e and Supplementary 5a**).

2. Additional demonstration of CHIP+PKAc interaction in other cells/tissues beyond what is shown here would be welcome: bone cells? adrenal?

AR: We have identified complex formation of affinity isolated macromolecular PKA complexes with CHIP in a collection of cell lines and also glioblastoma tissue. We highlight this finding now in a revised Fig. 1 (+legend) showing that indeed we identify this complex in A375 melanoma cells, in KM12 and SW480 colon cancer cells, in osteosarcoma cells (U2OS) and in patient tissue (glioblastoma) (**please, see lines 141 and 142, and new Fig. 1e**).

3. How does the ubiquitylation of PKAc at lysine 310 affect interaction with PKI? it should be explored

AR: We performed the suggested experiments. To cut it short. We don't see an effect of CHIP coexpression on PKAc:PKI interaction. We present this data in the **new Supplementary Fig. 5b.**

Other minor comments:

1. In genetics, these ataxias are abbreviated as SCAs (not SCAR).

AR: The reviewer is correct when referring to autosomal dominant SCAs. However, for the autosomal recessive variant reported in the manuscript the correct name is SCAR16.

2. The LTP work is somewhat weak and I wonder whether it should remain here, especially in light of the additional work suggested above.

AR: We believe that the data on LTP in CHIP KO mice further supports the biological relevance of CHIP in the PKAc-mediated signaling network *in vivo*. For this reason, we would leave the data in the main figures. However, if necessary, we can move these data to the Supplementary Section.

REVIEWERS' COMMENTS:

Reviewer #2 (Remarks to the Author):

The authors have carefully addressed by concerns and I only have a couple of minor suggestions:

Line 347: our results indicate that CHIP and HSP70 activity are essential elements for cAMP-dependent synaptic plasticity processes.

Comment: When the effects on chemLTP are this small and variable you should not call them essential elements. I suggest “our results indicate that CHIP and HSP70 are modulators of cAMP-dependent synaptic plasticity.”

Line 308: Further, VER155008 blocked the induction of CREB phosphorylation initiated by FSK, or isoproterenol (an agonist of the cAMP-linked beta2-adrenergic receptor) exposure pathway.

Comment: The Hsp70 inhibitor induced pCREB on its own so the authors might say that it prevents further induction of pCREB but it really does not block the induction of CREB phosphorylation. There is still no data that I can find for an iso effect on CREB phosphorylation. In Figure 6d the authors should indicate the addition of H89.

Reviewer: Stanley McKnight

Reviewer #3 (Remarks to the Author):

The revised manuscript is significantly improved.

The LTP data may be added in the supplementary material.

REVIEWERS' COMMENTS:

<u>Reviewer #2 (Remarks to the Author)</u>:

The authors have carefully addressed by concerns and I only have a couple of minor suggestions.

Author Response (AR): We wish to thank the Reviewer for his constructive and insightful comments that helped to improve the quality of the manuscript, and in finding that 'we have carefully address his concerns'.

Please, see below are responses:

Line 347: our results indicate that CHIP and HSP70 activity are essential elements for cAMP-dependent synaptic plasticity processes.

Comment: When the effects on chemLTP are this small and variable you should not call them essential elements. I suggest "our results indicate that CHIP and HSP70 are modulators of cAMP-dependent synaptic plasticity."

Author Response (AR): As suggested, we have changed the above sentence to 'our results indicate that CHIP and HSP70 are **modulators** of cAMP-dependent synaptic plasticity' (line 337).

Line 308: Further, VER155008 blocked the induction of CREB phosphorylation initiated by FSK, or isoproterenol (an agonist of the cAMP-linked beta2-adrenergic receptor) exposure pathway.

Comment: The Hsp70 inhibitor induced pCREB on its own so the authors might say that it prevents further induction of pCREB but it really does not block the induction of CREB phosphorylation.

Author Response (AR): As suggested, we have changed the above sentence to 'Hsp70 inhibitor **prevented** further induction of CREB phosphorylation (please, see line 299).

Comment: There is still no data that I can find for an iso effect on CREB phosphorylation.

Author Response (AR): We apologize for the confusion. We have corrected the text accordingly.

Comment: In Figure 6d the authors should indicate the addition of H89.

Author Response (AR): We apologize for the inconvenient. During the assembly of the figure, the label to Fig.6d was mistakenly lost. Now, we added H89 to the figure panel.

<u>Reviewer #3 (Remarks to the Author)</u>:

The revised manuscript is significantly improved.

Author Response (AR): Many thanks to the Reviewer for the constructive and insightful comments during the revision, and in finding our revised manuscript 'significantly improved'. Please, see below our responses:

The LTP data may be added in the supplementary material.

Author Response (AR): We moved the LTP data in the **new Supplementary Fig. 9**
